# Steroid hormone-related polymorphisms associate with the development of bone erosions in rheumatoid arthritis and help to predict disease progression: Results from the REPAIR consortium

**DOI:** 10.1038/s41598-019-51255-0

**Published:** 2019-10-15

**Authors:** Jose M. Sánchez-Maldonado, Rafael Cáliz, Luz Canet, Rob ter Horst, Olivier Bakker, Alfons A. den Broeder, Manuel Martínez-Bueno, Helena Canhão, Ana Rodríguez-Ramos, Carmen B. Lupiañez, María José Soto-Pino, Antonio García, Eva Pérez-Pampin, Alfonso González-Utrilla, Alejandro Escudero, Juana Segura-Catena, Romana T. Netea-Maier, Miguel Ángel Ferrer, Eduardo Collantes-Estevez, Miguel Ángel López Nevot, Yang Li, Manuel Jurado, João E. Fonseca, Mihai G. Netea, Marieke J. H. Coenen, Juan Sainz

**Affiliations:** 1 Genomic Oncology Area, GENYO, Centre for Genomics and Oncological Research: Pfizer/University of Granada/Andalusian Regional Government, PTS Granada, Granada, Spain; 2 Instituto de Investigación Biosanataria IBs.Granada, Granada, Spain; 3 Rheumatology department, Virgen de las Nieves University Hospital, Granada, Spain; 4 Department of Internal Medicine and Radboud Center for Infectious Diseases, Radboud University Nijmegen Medical Center, Nijmegen, The Netherlands; 5 Department of Genetics, University of Groningen, University Medical Center Groningen, Groningen, The Netherlands; 6 Department of Rheumatology, Radboud University Medical Center, Nijmegen, The Netherlands; 7 Area of Genomic Medicine, GENYO, Centre for Genomics and Oncological Research: Pfizer/University of Granada/Andalusian Regional Government, Granada, Spain; 8 CEDOC, EpiDoC Unit, NOVA Medical School and National School of Public Health, Universidade Nova de Lisboa, Lisbon, Portugal; 9 Rheumatology Unit, University Hospital of Santiago de Compostela, Santiago de Compostela, Spain; 10 Rheumatology department, Reina Sofía Hospital/IMIBIC/University of Córdoba, Córdoba, Spain; 11 Immunology department, Virgen de las Nieves University Hospital, Granada, Spain; 12 Rheumatology and Metabolic Bone Diseases Department, Hospital de Santa Maria, CHLN, Lisbon, Portugal; 13 Rheumatology Research Unit, Instituto de Medicina Molecular, Faculty of Medicine, University of Lisbon, Lisbon Academic Medical Center, Lisbon, Portugal; 14 Department for Immunology & Metabolism, Life and Medical Sciences Institute (LIMES), University of Bonn, 53115 Bonn, Germany; 15 Department of Human Genetics, Radboud University Medical Center, Radboud Institute for Health Sciences, Nijmegen, The Netherlands

**Keywords:** Prognostic markers, Rheumatoid arthritis

## Abstract

Here, we assessed whether 41 SNPs within steroid hormone genes associated with erosive disease. The most relevant finding was the rheumatoid factor (RF)-specific effect of the *CYP1B1*, *CYP2C9*, *ESR2*, *FcγR3A*, and *SHBG* SNPs to modulate the risk of bone erosions (*P* = 0.004, 0.0007, 0.0002, 0.013 and 0.015) that was confirmed through meta-analysis of our data with those from the DREAM registry (*P* = 0.000081, 0.0022, 0.00074, 0.0067 and 0.0087, respectively). Mechanistically, we also found a gender-specific correlation of the *CYP2C9*_rs1799853T/T_ genotype with serum vitamin D3 levels (*P* = 0.00085) and a modest effect on IL1β levels after stimulation of PBMCs or blood with LPS and PHA (*P* = 0.0057 and *P* = 0.0058). An overall haplotype analysis also showed an association of 3 *ESR1* haplotypes with a reduced risk of erosive arthritis (*P* = 0.009, *P* = 0.002, and *P* = 0.002). Furthermore, we observed that the *ESR2*, *ESR1* and *FcγR3A* SNPs influenced the immune response after stimulation of PBMCs or macrophages with LPS or Pam3Cys (P = 0.002, 0.0008, 0.0011 and 1.97•10^−7^). Finally, we found that a model built with steroid hormone-related SNPs significantly improved the prediction of erosive disease in seropositive patients (*P*_RF+_ = 2.46•10^−8^) whereas no prediction was detected in seronegative patients (*P*_RF−_ = 0.36). Although the predictive ability of the model was substantially lower in the replication population (*P*_RF+_ = 0.014), we could confirm that *CYP1B1* and *CYP2C9* SNPs help to predict erosive disease in seropositive patients. These results are the first to suggest a RF-specific association of steroid hormone-related polymorphisms with erosive disease.

## Introduction

Rheumatoid arthritis (RA) is a chronic and disabling chronic immune-mediated inflammatory disease that affects approximately 1% of the worldwide population^1^. Although the etiology of this autoimmune disease remains largely unknown, family- and population-based genome-wide association studies (GWAS) have consistently demonstrated that RA has a strong inherited component that influences not only the predisposition to develop the disease^2–5^. Even though there are no relevant predictors for treatment response in RA^6^, recent studies have suggested that inherited genetic factors might influence the response to both classical disease-modifying anti-rheumatic or biological drugs^7–11^ and even disease progression^12–14^. From epidemiological studies, it has also been proposed that in addition to inherited factors and environmental factors certain hormonal events might help to promote the onset of the disease^15–17^. It has been suggested that concentrations of steroid hormones and circulating immunocomplexes (CICs) in the synovial fluid and cartilage may contribute to promote gender-specific inflammatory responses, a differently controlled and sustained production of autoantibodies between men and women^18–20^ and a different release of a wide range of cytokines and pro-inflammatory mediators that trigger sustained and chronic inflammatory responses^21–23^. However, there is still controversy about the effect of steroid hormones on the risk of developing RA or disease progression since the administration of different hormone replacement therapies or the use of oral contraceptives has not been associated with the risk of RA and its progression in most of the epidemiological studies conducted to date^24–27^.

Some authors have hypothesized that these controversial results might be, at least in part, due to the presence of certain factors such as the HLA-DRB1 shared epitope or specific autoantibodies such as antibodies to cyclic citrullinated peptide (anti-CCP)^28,29^. Furthermore, there are contradictory results concerning to the role of steroid hormones in the modulation of immune responses. Some studies have reported, for instance, that vitamin D3 has immunomodulatory properties that may influence autoimmune disease risk and disease progression^30^ whereas some other have suggested that estrogens can induce both tolerogenic and pro-inflammatory responses at multiple levels and that this may result in remarkable sex differences on immune function. Under appropriate circumstances, estrogens may inhibit Th_1_- and Th_17_-mediated immune functions^31,32^ and stimulate Treg cell development^33^ and the activation of Th_2_-mediated immune responses. However, it has been also demonstrated that estrogens may induce pro-inflammatory responses. For instance, it has been suggested that estrogens influence *FcγR3A* mRNA gene expression and induce the *FcγR3A*-mediated release of tumor necrosis factor (TNF) and IL1β from monocytes^34^, thereby modulating degranulation, antibody-dependent cellular cytotoxicity (ADCC), transcription of cytokine genes, rapid release of inflammatory mediators and reactive oxygen species, and phagocytosis^35–37^. Considering the role of FcγR proteins in modulating autoimmune responses but also the plausibility of a gender-specific effect of estrogens to modulate immune responses, we aimed at analyzing whether the presence of single nucleotide polymorphisms (SNPs) within steroid hormone signaling (*ESR1*, *ESR2*, *NR1I2*, *PGR*, *and SHBG*), phase I- and II-metabolizing enzyme (*CYP1A1*, *CYP1A2*, *CYP1B1*, *CYP17A1*, *CYP2C9*, *CYP2C19*, *CYP3A4*, *GSTP1*, *HSD17B1* and *SULT1A1*) and Fc gamma receptor (*FcγR3A* and *FCGR2A*) genes influence disease progression in RA. We assessed whether 41 potentially functional SNPs within these genes are associated with the risk of developing erosive disease and whether the effect of the SNPs on disease progression was modified by the presence of rheumatoid factor (RF) or anti-CCP. In order to confirm the consistency of our results, we performed fixed-effect meta-analyses with data from the DREAM registry. Finally, we also evaluated whether selected polymorphisms correlated with steroid hormone and cytokine levels and whether genotyping of selected SNPs might help us to improve the prediction of the appearance of bone erosions.

## Patients and Methods

### Study population

This retrospective cohort study included 816 RA patients ascertained through the REPAIR consortium (567 showing erosive disease and 249 without bone erosions). RA patients fulfilled the 1987 revised American College of Rheumatology (ACR)^38^ and the ACR/EULAR 2010 classification criteria^39^. A detailed description of the population has been reported elsewhere^40–42^. Briefly, 518 RA patients were recruited at the department of Rheumatology of the Virgen de las Nieves Hospital (Granada, Spain), the Reina Sofia Hospital (Córdoba, Spain), and the University Clinical Hospital of Santiago de Compostela (Santiago de Compostela, Spain). Two hundred and ninety-eight RA patients were additionally recruited from the Santa Maria Hospital–CHLN (Biobanco-IMM; Lisbon Academic Medical Centre, Lisbon, Portugal). The study was performed according to the Helsinki Declaration. All participants were of European ancestry and gave their written informed consent to participate in the study. The Ethics committee of each participant institution approved the study protocol: Virgen de las Nieves University Hospital (2012/89); Santa Maria Hospital-CHLN (CE 877/121.2012); University Clinical Hospital of Santiago de Compostela (2013/156). A detailed description of demographic and clinical variables of this population is included in Table 1.

**Table 1:** Demographic and clinical characteristics of RA patients included in the discovery and replication cohorts.

	Discovery Population All patients	Discovery Population Patients with erosive disease	Discovery Population Patients without erosive disease	P-value	Replication Population All patients	Replication Population Patients with erosive disease	Replication Population Patients without erosive disease	P-value
Demographic characteristics	(n = 816)	(n = 567)	(n = 249)		(n = 436)	(n = 307)	(n = 129)	
Age (years)	59.32 ± 13.11	59.66 ± 12.47	58.95 ± 14.30	0.50	53.59 ± 12.84	53.63 ± 10.17	53.47 ± 13.30	0.90
Sex ratio (female/male)	3.74 (644/172)	4.4 (462/105)	2.71 (182/67)	0.007	2.06 (294/143)	2.13 (209/98)	1.87 (84/45)	0.55
**Clinical assessment**								
Percentage of patients with RF positivity*	571 (70.58)	409 (72.52)	162 (66.12)	0.07	328 (77.72)	235 (78.33)	93 (76.23)	0.64
Percentage of ACPA-positive patients*	490 (72.80)	354 (74.21)	136 (69.39)	0.20	90 (58.06)	64 (58.72)	26 (61.91)	0.72
DAS28 at baseline	5.63 ± 2.40	5.57 ± 1.18	5.79 ± 4.17	0.41	5.24 ± 1.27	5.27 ± 1.23	5.16 ± 1.36	
Disease follow-up (years)	18.30 ± 9.34	19.43 ± 9.00	17.80 ± 14.44	0.10	9.00 ± 9.87	9.17 ± 10.17	8.58 ± 9.09	
Percentage of RA patients having erosive disease	567 (69.49)	567 (100.0)	0 (0.0)	—	307 (70.41)	307 (100.0)	0 (0.0)	—
Percentage of RA patients with biologic treatments	632 (77.45)	448 (79.01)	184 (73.90)	0.11	436 (100.00)	307 (100.0)	129 (100.0)	1.00
**DMARDs**								
Methotrexate^∂^	603 (79.24)	415 (79.20)	188 (79.32)	0.97	314 (75.48)	226 (77.13)	88 (71.54)	0.23
**First biological treatment**								
Infliximab (%)	271 (42.88)	221 (44.02)	50 (37.88)	0.25	106 (24.31)	79 (25.73)	27 (20.93)	0.29
Etanercept (%)	176 (27.85)	134 (26.69)	42 (32.30)	0.20	101 (23.16)	72 (23.45)	29 (22.48)	0.83
Adalimumab (%)	157 (24.84)	126 (25.10)	31 (23.85)	0.77	229 (52.52)	156 (50.81)	73 (56.59)	0.27
Golimumab (%)	11 (1.74)	7 (01.39)	4 (03.08)	0.19	—	—	—	—
Abatacep (%)	5 (0.79)	4 (00.80)	1 (00.77)	0.98	—	—	—	—
Tocilizumab (%)	4 (0.63)	2 (00.40)	2 (01.52)	0.14	—	—	—	—
Rituximab (%)	8 (1.27)	8 (01.59)	0 (00.00)	—	—	—	—	—

Data are means ± standard deviation or n (%). Abbreviations: RF, rheumatoid factor; ACPA: anti-citrullinated protein antibodies; DAS28, disease activity score; DMARDs, disease-modifying anti-rheumatic drugs. P < 0.05 in bold.

*RF and anti-CCP data were available in 809 and 673 RA patients in the discovery population, respectively.

*RF and anti-CCP data were available in 564 and 477 RA patients with erosive disease in the discovery population, respectively.

*RF and anti-CCP data were available in 245 and 196 RA patients without erosive disease in the discovery population, respectively

*RF and anti-CCP data were available in 422 and 151 RA patients in the replication population, respectively.

*RF and anti-CCP data were available in 300 and 109 RA patients with erosive disease in the replication population, respectively.

*RF and anti-CCP data were available in 122 and 42 RA patients without erosive disease in the replication population, respectively.

^*∂*^Information about methotrexate treatment was available in 761 and 416 patients in the discovery and replication populations, respectively.

^*∂*^Information about methotrexate treatment was available in 524 and 293 patients with erosive disease in the discovery and replication populations, respectively.

^*∂*^Information about methotrexate treatment was available in 237 and 123 patients without erosive disease in the discovery and replication populations, respectively.

### Bone erosions

Bone erosions were visible in plain radiographs and defined as the interruption of the cortical bone surface within the joint region or underlying the cartilage^43,44^. Bone erosions were then coded as present or absent. All radiographs were assessed by, at least, an experienced radiologist or rheumatologist.

### SNP selection and genotyping

We conducted an extensive literature search concerning the mechanism of action of estrogen and progesterone receptor, hormone transporter, and hormone-metabolizing enzyme genes was performed to select candidate genes that might affect the risk of developing bone erosions. SNPs were assessed on the basis of NCBI data and were selected according to their known or putative functional consequences, i.e. their modifying influence on the structure of proteins, transcription level, or alternative splicing mechanisms. SNPs within the same gene were also selected on the basis of linkage disequilibrium (LD) data. In total, 41 SNPs in 17 genes were selected for this study (Table 2).

**Table 2:** Selected SNPs within steroid hormone-related genes.

Gene	Chr.	dbSNP rs#	Nucleotide substitution	Effect-allele	Amino acid substitution
*CYP1A1*	15	rs1799814	A/C	A	T461N
*CYP1A2*	15	rs762551	A/C	C	intronic
*CYP1B1*	2	rs1800440	A/G	G	N453T
*CYP1B1*	2	rs1056836	C/G	G	L432V
*CYP1B1*	2	rs10012	C/G	G	R48G
*CYP2C9*	10	rs1799853	C/T	T	R144C
*CYP2C9*	10	rs1057910	A/C	C	I359L
*CYP2C19*	10	rs12248560	C/T	T	Near gene
*CYP2C19*	10	rs4244285	A/G	A	P227P
*CYP3A4*	7	rs2740574	A/G	G	Near gene
*CYP3A4*	7	rs11773597	C/G	C	Near gene
*CYP17A1*	10	rs743572	A/G	G	5′-UTR
*ESR1*	6	rs851984	C/T	T	intronic
*ESR1*	6	rs2881766	G/T	G	intronic
*ESR1*	6	rs2071454	G/T	G	5′-UTR
*ESR1*	6	rs2077647	A/G	G	S10S
*ESR1*	6	rs827421	C/T	C	intronic
*ESR1*	6	rs2234693	C/T	C	Intronic
*ESR1*	6	rs9340799	A/G	G	intronic
*ESR1*	6	rs1801132	C/G	G	P325P
*ESR1*	6	rs3798577	C/T	C	3′-UTR
*ESR1*	6	rs910416	C/T	T	Near gene
*ESR2*	14	rs1255998	C/G	G	3′-UTR
*ESR2*	14	rs928554	A/G	G	3′-UTR
*ESR2*	14	rs4986938	T/C	T	3′-UTR
*ESR2*	14	rs1271572	G/T	T	Near gene
*FcγR2A*	1	rs1801274	A/G	G	H131R
*FcγR3A*	1	rs396991	A/C	C	V158F
*GSTP1*	11	rs1695	A/G	G	I105V
*GSTP1*	11	rs1138272	C/T	T	A114V
*HSD17B1*	17	rs605059	C/T	T	G313S
*NR1I2*	3	rs2276706	A/G	A	Near gene
*NR1I2*	3	rs1464603	C/T	C	intronic
*NR1I2*	3	rs6785049	A/G	G	intronic
*NR1I2*	3	rs2276707	C/T	T	intronic
*NR1I2*	3	rs1054191	A/G	A	3′-UTR
*PGR*	11	rs1042838	C/A	A	V660L
*PGR*	11	rs1379130	A/G	A	G393G
*PGR*	11	rs518162	A/G	A	5′-UTR
*SHBG*	17	rs6259	A/G	A	D356N
*SULT1A1*	16	rs9282861	A/G	A	R213H

Abbreviations: SNP, single nucleotide polymorphism; MAF, minor allele frequency; UTR, untranslated region.

Genotyping of selected steroid hormone-related SNPs was performed at GENYO (Granada, Spain) using KASPar® probes with the exception of the *FcγR3A*_rs396991_ and *FC*γ*R2A*_rs1801274_ SNPs that were determined using TaqMan® SNP Genotyping Assays (Life Technologies, Carlsbad, CA, USA). Both KASPar® and Taqman® assays were assayed according to the manufacturer’s specifications for a 384-well plate format. Genomic DNA was extracted from peripheral blood mononuclear cells (PBMCs) using Qiagen Mini kit (Qiagen, Valencia, CA, USA) and PCR products were analyzed with ABI Prism 7900HT detection system using the SDS 2.4 software (Applied Biosystems). Five percent of samples were included in the PCR plates as duplicates and concordance between the analyzed original and duplicated samples was >99.0%.

### Statistical analysis

Hardy-Weinberg Equilibrium (HWE) was assessed in the control group by a chi-square (χ^2^) test. Logistic regression analysis adjusted for age, sex and country of origin was used to assess the main effect of the selected SNPs on disease progression (defined as the presence of bone erosions). RF− and anti-CCP-stratified analyses were also carried out and we included RF as interaction term in the overall logistic regression analysis to evaluate whether there was any statistically significant effect modification by these factors. Haplotype analysis using the same variables for adjustment was conducted using the R package Haplo.stats^45^. In order to facilitate eventual meta-analyses, the major allele was set as reference allele. All tests were conducted using the statistical software STATA (v.12) and R (http://www.r-project.org). In order to account for multiple testing, we set a *P* value of 0.00074 as significance study-wide threshold. The *P* value was calculated considering the number of independent polymorphisms analyzed (n = 34, MeffLi method)^46^ but also the number of inheritance models tested (dominant and recessive).

### Linkage disequilibrium (LD) and haplotype analysis

We performed haplotype frequency estimation and haplotype association analysis adjusted for age, sex and country of origin using the haplo.stats^45^. Haplotype frequencies were determined using the Expectation-maximization (EM) algorithm and haplotypes were reconstructed using SNPtools^47^ and Haploview^48^. Block structures were determined according to the method of Gabriel *et al*.^49^ (Supplementary Fig. 1).

### Replication population and meta-analysis

With the aim of assessing the consistency of the overall and RF-specific associations between SNPs and the risk of developing bone erosions, we genotyped the most interesting markers in a replication population from the DREAM registry consisting of 436 RA patients (307 RA patients with bone erosions and 129 patients without erosive disease). Demographic and clinical parameters of this population are also included in Table 1. We performed a meta-analysis of the data obtained in the discovery population with those from the DREAM registry and we pooled the Odds Ratios (ORs) for the most interesting polymorphisms using a fixed-effect model. Coefficients with a *P*-value ≤ 0.05 were considered significant. I^2^ statistic was used to assess heterogeneity between studies.

### Functional analysis of the estrogen-related variants

Cytokine stimulation experiments were conducted in the 500 Functional Genomics (500FG) cohort from the Human Functional Genomics Project (HFGP; http://www.humanfunctionalgenomics.org/), which was designed to determine the influence of genomic variation on the variability of immune responses. The HFGP study was approved by the Arnhem-Nijmegen Ethical Committee (no. 42561.091.12) and biological specimens were collected after informed consent was obtained. We investigate whether any of the 41 estrogen-related SNPs correlated with cytokine levels (IFNγ, IL1β, IL6, TNFα, IL17, and IL22) after the stimulation of peripheral blood mononuclear cells (PBMCs), macrophages or whole blood from 408 healthy subjects with LPS (1 or 100 ng/ml), PHA (10 μg/ml), and Pam3Cys (10 μg/ml). After log transformation, linear regression analyses adjusted for age and sex were used to determine the correlation of selected SNPs with cytokine expression quantitative trait loci (cQTLs). All analyses were performed using R software (http://www.r-project.org/). In order to account for multiple comparisons, we used a significant threshold of 0.00025 (0.05/34 independent SNPs × 6 cytokines).

Details on PBMCs isolation, macrophage differentiation and stimulation assays have been reported elsewhere^50–52^. Briefly, PBMCs were washed twice in saline and suspended in medium (RPMI 1640) supplemented with gentamicin (10 mg/mL), L-glutamine (10 mM) and pyruvate (10 mM). PBMC stimulations were performed with 5 × 10^5^ cells/well in round-bottom 96-wells plates (Greiner) for 24 hours in the presence of 10% human pool serum at 37 °C and 5% CO_2_. Supernatants were collected and stored in −20 °C until used for ELISA. LPS (100 ng/ml), PHA (10 μg/ml) and Pam3Cys (10 μg/ml) were used as stimulators for 24 or 48 hours. Whole blood stimulation experiments were conducted using 100 μl of heparin blood that was added to a 48 well plate and subsequently stimulated with 400 μl of LPS and PHA (final volume 500 ul) for 48 hours at 37 °C and 5% CO_2_. Supernatants were collected and stored in −20 °C until used for ELISA. Concentrations of human IFNγ, IL1β, IL6, TNFα, IL17, and IL22 were determined using specific commercial ELISA kits (PeliKine Compact, Amsterdam, or R&D Systems), in accordance with the manufacturer’s instructions.

Once we assessed the correlation of estrogen-related SNPs with cytokine levels, we used the HaploReg SNP annotation tool (http://www.broadinstitute.org/mammals/haploreg/haploreg.php) to further investigate the functional consequences of each specific variant. We also assessed whether any of the potentially interesting markers correlated with mRNA expression levels of their respective genes using data from public eQTL browsers (GTex portal; www.gtexportal.org/home/ and Blood eQTL browser; https://genenetwork.nl/bloodeqtlbrowser/).

### Correlation between steroid hormone levels and hormone-related SNPs

We also measured serum levels of seven steroid hormones (androstenedione, cortisol, 11-deoxy-cortisol, 17-hydroxy progesterone, progesterone, testosterone and 25 hydroxy vitamin D3) in the 500FG cohort, which includes 531 healthy subjects. Complete protocol details of steroid hormone measurements have been reported elsewhere^52^. Hormone levels and genotyping data were available for a total of 406 subjects.

After log-transform, correlation between steroid hormone levels and steroid hormone-related SNPs was evaluated by linear regression analysis adjusted for age and sex (or for age when men and women were analysed separately). In order to avoid a possible bias, we excluded from the analysis those subjects that were using oral contraceptives or those subjects in which this information was not available. A total of 279 healthy subjects (107 women and 272 men) were finally available for analysis. Significance threshold was set to 0.00021 considering the number of independent SNPs tested (n = 34) and the number of hormones determined (n = 7).

### Predictive models and discriminative accuracy

The value of steroid hormone-related variants for prediction of prognosis and disease progression in seropositive and seronegative RA patients was assessed using stepwise logistic regression. Models were built including demographic variables (age and sex) and genetic polymorphisms that showed significant associations with erosive disease in the single-SNP analysis (*P* < 0.05). The genetic model was then compared with the reference model including demographic variables. The area under the curve (AUC) of a receiver operating characteristic (ROC) curve analysis and −2 log likelihood ratio (LR) tests were used to assess whether the genetic models fitted significantly better the data compared to their respective reference models. Finally, we run randomization tests to confirm whether the improved predictive ability of each genetic model was consistent after 50.000 iterations. All tests were conducted using R software (http://www.r-project.org/).

### Ethics approval

The study was approved by the ethical review committee of each participant institution (Virgen de las Nieves University Hospital, Granada, Spain; Reina Sofia Hospital, Córdoba, Spain; University Clinical Hospital of Santiago de Compostela, Santiago de Compostela, Spain; Biobanco-IMM, Lisbon Academic Medical Centre, Portugal. Cytokine stimulation experiments and hormone analysis were approved by the Arnhem-Nijmegen Ethical Committee.

## Results

Erosive RA patients had a similar age than those patients without bone erosions (59.66 ± 12.47 vs. 58.95 ± 14.30, *P* = 0.50) and had a significantly higher female to male ratio (462/105 = 4.4 vs. 182/67 = 2.71, *P* = 0.007; Table 1). Overall, the percentage of RA patients with positive RF and anti-CCP was 70.58% and 72.80% respectively, and these percentages were slightly higher among those patients with bone erosions (72.52% and 74.21%) than in those without erosive disease (66.12% and 69.39%). The mean of disease follow-up was 18.30 years whereas the mean of DAS28 was 5.63. Six hundred and three patients received methotrexate (79.24%) and 632 patients (77.45%) were treated with biologic therapies. With the exception of gender, none of the demographical or clinical variables significantly differ between patient with and without erosive disease (Table 1).

### Association of steroid hormone-related polymorphisms with the risk of having bone erosions

Selected polymorphisms did not show deviation from HWE in the control population (patients without erosive disease; *P* > 0.001). Logistic regression analysis adjusted for age, gender and country of origin revealed that carriers of the *ESR2*_rs1271572T/T_ genotype tended to have a decreased risk of developing erosive disease than those subjects carrying the G allele (OR = 0.55, *P* = 0.004: Table 3). Although the association of the *ESR2*_rs1271572T/T_ genotype with a decreased risk of having bone erosions remained only marginally significant after correction for multiple testing, we found a significant RF-specific effect of this SNP to modulate the risk of having erosive disease. Seropositive patients carrying the *ESR2*_rs1271572T/T_ genotype had a significantly reduced risk of developing erosive disease (OR = 0.38, *P* = 0.0002) whereas an opposite but not significant effect was found in seronegative patients (OR = 1.08, *P* = 0.83; *P*_Int_ = 0.018; Table 3). Importantly, the association of the *ESR2*_rs1271572_ SNP with a reduced risk of developing bone erosions in seropositive patients remained significant after correction for multiple testing (P < 0.00074). Although the association was not replicated in the DREAM cohort, the meta-analysis of our data and those from the DREAM registry, including 1252 RA patients, confirmed that seropositive patients carrying the *ESR2*_rs1271572T/T_ genotype had a decreased risk of developing erosive disease (OR_RF+_ = 0.52, *P* = 0.00074) whereas a totally opposite but not statistically significant effect was found in seronegative patients (OR_RF−_ = 1.28, *P* = 0.42; *P*_Het_ = 0.33; Table 4). No significant anti-CCP effect modification was found for this SNP to modulate the risk of developing bone erosions (*P*_Int_ = 0.11; Supplementary Table 1), which suggest that *ESR2* locus might play a relevant role in determining disease progression in a RF-dependent manner. In agreement with these results, we found a RF-specific effect of the *ESR2*_CGTA_ haplotype to determine the risk of developing erosive disease. Seropositive RA patients carrying the *ESR2*_CGTA_ haplotype (and, therefore, not harboring the *ESR2*_rs1271572_ protective allele) showed an increased risk of developing bone erosions (OR_RF+_ = 1.63, *P* = 0.0051) whereas an opposite but not significant effect was detected in seronegative patients (OR_RF−_ = 0.93, *P* = 0.99; Supplementary Table 2). An overall haplotype analysis including 1252 RA patients from the discovery and replication populations confirmed the RF-specific association of the *ESR2*_CGTA_ haplotype with an increased risk of developing bone erosions (OR_RF+_ = 1.44, 95%CI 1.13–1.84; *P* = 0.0036 and OR_RF−_ = 0.89, 95%CI 0.62–1.26; *P* = 0.51). According to publicly available gene expression datasets (GTex portal and Haploreg), the *ESR2*_rs1271572_ variant strongly correlate with *ESR2* mRNA expression levels in whole peripheral blood (*P* = 3.1•10^−9^) but also in primary B cells, lymphoblastoid cell lines (from *P* = 1.98•10^−6^ to *P* = 3.47•10^−10^) and several tissues (ranging from *P* = 2.60•10^−5^ to *P* = 8.33•10^−23^; Supplementary Table 3). Intriguingly, a similar level of correlation with gene expression was found for other variants belonging to the *ESR2*_CGTA_ haplotype (Supplementary Table 3), which strongly suggested that the *ESR2*_rs1271572_ SNP or *ESR2*_CGTA_ haplotype might represent an eQTL for *ESR2*.

**Table 3:** Overall and RF-specific associations of estrogen-related polymorphisms and risk of developing erosive disease.

Gene	SNP ID	Effect allele	Overall (n = 816)		RF-positive patients (n = 571)		RF-negative patients (n = 238)		*P*_*Interaction*_
			OR (95% CI)^†^	*P*	OR (95% CI)^†^	*P*	OR (95% CI)^†^	*P*	
*CYP1A1*	rs1799814	A	0.85 (0.52–1.39)	0.52	0.82 (0.43–1.54)	0.53	0.98 (0.44–2.18)	0.96	0.54
*CYP1A2*	rs762551	C	0.91 (0.66–1.25)	0.57	0.72 (0.48–1.09)	0.12	1.37 (0.80–2.34)	0.25	0.059
*CYP1B1*	rs1800440	G	1.06 (0.76–1.46)	0.74	1.12 (0.74–1.69)	0.60	0.97 (0.55–1.69)	0.90	0.50
*CYP1B1*	rs1056836	G	0.92 (0.64–1.32)	0.66	1.04 (0.67–1.63)	0.85	0.66 (0.34–1.26)	0.21	0.35
*CYP1B1*	rs10012	G	0.62 (0.37–1.04)^§^	0.071	**0**.**42 (0**.**23–0**.**76)**^**§**^	**0**.**004**	1.70 (0.56–5.17)^§^	0.35	**0**.**031**
*CYP2C9*	rs1799853	T	0.45 (0.20–1.02)^§^	0.056	**0**.**16 (0**.**06–0**.**46)**^**§**^	**0**.**0007**	2.71 (0.53–13.8)^§^	0.23	**0**.**003**
*CYP2C9*	rs1057910	C	1.68 (0.98–2.89)	0.059	**2**.**63 (1**.**15–6**.**03)**	**0**.**012**	1.28 (0.58–2.81)	0.54	0.20
*CYP2C19*	rs12248560	T	1.04 (0.75–1.44)	0.83	1.09 (0.72–1.65)	0.70	0.96 (0.54–1.70)	0.89	0.55
*CYP2C19*	rs4244285	A	1.00 (0.69–1.44)	0.99	1.02 (0.64–1.61)	0.94	0.90 (0.47–1.70)	0.74	0.79
*CYP3A4*	rs2740574	G	1.57 (0.90–2.74)	0.11	**3**.**12 (1**.**29–7**.**55)**	**0**.**004**	0.70 (0.29–1.66)	0.42	**0**.**021**
*CYP3A4*	rs11773597	C	1.19 (0.77–1.84)	0.43	1.33 (0.74–2.38)	0.35	1.00 (0.50–2.03)	0.99	0.65
*CYP17A1*	rs743572	G	0.92 (0.66–1.28)	0.63	0.99 (0.65–1.50)	0.97	0.82 (0.46–1.45)	0.49	0.62
*ESR1*	rs851984	T	1.07 (0.78–1.46)	0.68	0.82 (0.55–1.24)	0.35	1.56 (0.91–2.68)	0.10	0.078
*ESR1*	rs2881766	G	1.00 (0.72–1.39)	0.99	1.08 (0.71–1.65)	0.71	0.83 (0.47–1.48)	0.54	0.47
*ESR1*	rs2071454	G	0.96 (0.65–1.42)	0.82	1.06 (0.65–1.75)	0.81	0.75 (0.38–1.49)	0.42	0.41
*ESR1*	rs2077647	G	0.92 (0.65–1.30)	0.64	0.70 (0.45–1.09)	0.11	1.46 (0.81–2.63)	0.21	**0**.**030**
*ESR1*	rs827421	C	0.95 (0.67–1.33)	0.75	0.80 (0.52–1.25)	0.33	1.21 (0.66–2.20)	0.54	0.18
*ESR1*	rs2234693	C	1.10 (0.78–1.55)	0.58	0.97 (0.62–1.51)	0.89	1.33 (0.73–2.40)	0.35	0.32
*ESR1*	rs9340799	G	0.97 (0.71–1.34)	0.87	0.78 (0.52–1.17)	0.23	1.42 (0.82–2.45)	0.21	0.052
*ESR1*	rs1801132	G	**0**.**71 (0**.**52–0**.**97)**	**0**.**034**	0.85 (0.57–1.27)	0.44	**0**.**53 (0**.**31–0**.**92)**	**0**.**025**	0.13
*ESR1*	rs3798577	C	1.21 (0.87–1.68)	0.27	1.39 (0.92–2.10)	0.12	1.12 (0.63–2.01)	0.69	0.50
*ESR1*	rs910416	T	0.84 (0.59–1.19)	0.33	0.75 (0.47–1.18)	0.21	0.91 (0.51–1.61)	0.74	0.68
*ESR2*	rs1255998	G	0.92 (0.64–1.33)	0.67	1.07 (0.66–1.72)	0.78	0.72 (0.39–1.34)	0.30	0.44
*ESR2*	rs928554	G	0.78 (0.52–1.17)^§^	0.23	**0**.**58 (0**.**35–0**.**96)**^**§**^	**0**.**035**	1.39 (0.70–2.78)^§^	0.35	**0**.**032**
*ESR2*	rs4986938	T	1.09 (0.79–1.51)	0.59	1.41 (0.93–2.11)	0.10	0.74 (0.42–1.29)	0.29	0.068
*ESR2*	rs1271572	T	**0**.**55 (0**.**37–0**.**82)**^**§**^	**0**.**004**	**0**.**38 (0**.**23–0**.**63)**^**§**^	**0**.**0002**	1.08 (0.54–2.14)^§^	0.83	**0**.**018**
*FcγR2A*	rs1801274	G	1.04 (0.72–1.51)	0.82	1.17 (0.74–1.86)	0.50	0.83 (0.43–1.61)	0.58	0.33
*FcγR3A*	rs396991	C	0.90 (0.64–1.27)	0.56	1.18 (0.76–1.81)	0.46	**0**.**45 (0**.**24–0**.**85)**	**0**.**013**	**0**.**028**
*GSTP1*	rs1695	G	1.05 (0.77–1.44)	0.76	1.16 (0.77–1.75)	0.47	0.70 (0.41–1.21)	0.20	0.26
*GSTP1*	rs1138272	T	1.32 (0.77–2.25)	0.31	1.70 (0.83–3.46)	0.13	0.71 (0.28–1.77)	0.46	0.24
*HSD17B1*	rs605059	T	1.12 (0.79–1.59)	0.54	1.24 (0.80–1.92)	0.33	1.12 (0.59–2.12)	0.74	0.82
*NR1I2*	rs2276706	A	1.01 (0.74–1.40)	0.93	0.96 (0.64–1.43)	0.83	1.18 (0.68–2.06)	0.55	0.47
*NR1I2*	rs1464603	C	1.16 (0.85–1.59)	0.35	1.14 (0.76–1.69)	0.53	1.45 (0.84–2.49)	0.18	0.54
*NR1I2*	rs6785049	G	0.92 (0.66–1.27)	0.60	0.95 (0.63–1.43)	0.81	1.03 (0.58–1.84)	0.92	0.93
*NR1I2*	rs2276707	T	1.03 (0.73–1.45)	0.87	0.80 (0.51–1.23)	0.31	1.62 (0.90–2.89)	0.11	0.064
*NR1I2*	rs1054191	A	0.83 (0.59–1.17)	0.30	1.02 (0.65–1.58)	0.94	0.58 (0.32–1.06)	0.076	0.12
*PGR*	rs1042838	A	0.76 (0.53–1.08)	0.13	0.74 (0.47–1.15)	0.18	0.86 (0.46–1.62)	0.64	0.99
*PGR*	rs1379130	A	0.92 (0.66–1.26)	0.59	1.02 (0.68–1.53)	0.93	0.79 (0.46–1.37)	0.40	0.59
*PGR*	rs518162	A	1.11 (0.75–1.64)	0.62	1.41 (0.83–2.39)	0.21	0.64 (0.33–1.25)	0.19	0.092
*SHBG*	rs6259	A	1.22 (0.83–1.78)	0.31	**1**.**87 (1**.**11–3**.**14)**	**0**.**015**	0.66 (0.35–1.23)	0.19	0.006
*SULT1A1*	rs9282861	A	1.04 (0.76–1.43)	0.79	0.86 (0.58–1.29)	0.46	1.38 (0.80–2.39)	0.25	0.13

Abbreviations: SNP, single nucleotide polymorphism; OR, odds ratio; CI, confidence interval.

Data on RF was available in 809 RA patients. Estimates were adjusted for age, sex and country of origin. P < 0.05 in bold.

^†^Estimates calculated according to a dominant model of inheritance.

^**§**^Estimates calculated according to a recessive model of inheritance.

**Table 4:** Replication of the most interesting associations between estrogen-related polymorphisms and risk of developing erosive disease (DREAM registry) and meta-analysis.

Gene	SNP ID	Effect allele	DREAM registry Overall (n = 436)			DREAM registry RF-positive patients (n = 328)			DREAM registry RF-negative patients (n = 94)			*P*_*Interaction*_
			OR (95% CI)^†^	*P*		OR (95% CI)^†^	*P*		OR (95% CI)^†^	*P*		
*CYP1B1*	rs10012	G	0.53 (0.27–1.06)^§^	0.073		**0**.**30 (0**.**13–0**.**70)**^**§**^	**0**.**0051**		5.97 (0.70–50.6)^§^	0.10		**0**.**012**
*CYP2C9*	rs1799853	T	NA (NA-NA)^§^	NA		NA (NA-NA)^§^	NA		NA (NA-NA)^§^	NA		NA
*CYP2C9*	rs1057910	C	1.89 (0.85–4.22)	0.095		**2**.**75 (1**.**03–7**.**35)**	**0**.**027**		0.54 (0.11–2.72)	0.47		0.073
*CYP3A4*	rs2740574	G	**0**.**36 (0**.**17–0**.**77)**	**0**.**008**		0.42 (0.17–1.07)	0.075		0.30 (0.08–1.22)	0.098		0.58
*ESR1*	rs1801132	G	**0**.**54 (0**.**31–0**.**96)**^§^	**0**.**035**		**0**.**39 (0**.**20–0**.**76)**^§^	**0**.**004**		1.43 (0.40–5.09)^§^	0.57		0.065
*ESR1*	rs9340799	G	0.69 (0.39–1.25)^§^	0.23		**0**.**42 (0**.**22–0**.**83)**^§^	**0**.**009**		**8**.**33 (1**.**02–67**.**8)**^**§**^	**0**.**011**		**0**.**008**
*ESR2*	rs1255998	G	**2**.**08 (1**.**17–3**.**69)**	**0**.**009**		1.82 (0.94–3.54)	0.065		**5**.**41 (1**.**15–25**.**4)**	**0**.**012**		0.19
*ESR2*	rs928554	G	**0**.**61 (0**.**37–1**.**00)**	**0**.**050**		0.59 (0.33–1.07)	0.075		0.51 (0.17–1.56)	0.22		0.79
*ESR2*	rs1271572	T	0.90 (0.56–1.47)^§^	0.68		0.78 (0.46–1.37)^§^	0.41		2.16 (0.64–7.27)^§^	0.19		0.32
*FcγR3A*	rs396991	C	0.74 (0.45–1.22)	0.25		0.78 (0.44–1.40)	0.41		0.53 (0.18–1.62)	0.27		0.58
*SHBG*	rs6259	A	0.86 (0.51–1.47)	0.59		1.17 (0.62–2.20)	0.63		**0**.**22 (0**.**07–0**.**71)**	**0**.**009**		**0**.**013**
**Gene**	**SNP ID**	**Effect allele**	**REPAIR + DREAM registry Meta-analysis Overall (n = 1252)**		**P**_**Het**_	**REPAIR + DREAM registry RF-positive patients (n = 899)**		**P**_**Het**_	**REPAIR + DREAM registry RF-negative patients (n = 332)**		**P**_**Het**_	
			**OR (95% CI)** ^†^	***P***		**OR (95% CI)** ^†^	***P***		**OR (95% CI)** ^†^	***P***		
*CYP1B1*	rs10012	G	**0**.**59 (0**.**39–0**.**88)**^§^	**0**.**011**	0.72	**0**.**38 (0**.**23–0**.**61)**^§^	**0**.**000081**	0.52	2.22 (0.83–5.95)^§^	0.11	0.31	
*CYP2C9*	rs1799853	T	NA (NA-NA)^**§**^	NA	NA	NA (NA-NA)^**§**^	NA	NA	NA (NA-NA)^**§**^	NA	NA	
*CYP2C9*	rs1057910	C	**1**.**74 (1**.**11–2**.**73)**	**0**.**015**	0.81	**2**.**68 (1**.**42–5**.**048)**	**0**.**0022**	0.95	1.08 (0.53–2.20)	0.83	0.34	
*CYP3A4*	rs2740574	G	0.94 (0.60–1.46)	0.77	**0**.**002**	1.19 (0.63–2.25)	0.59	**0**.**002**	0.55 (0.26–1.14)	0.11	0.30	
*ESR1*	rs1801132	G	**0**.**75 (0**.**58–0**.**97)**	**0**.**030**	0.52	0.78 (0.60–1.08)	0.13	0.51	0.72 (0.44–1.17)	0.19	**0**.**02**	
*ESR1*	rs9340799	G	0.90 (0.68–1.19)	0.45	0.31	**0**.**73 (0**.**53–1**.**00)**	**0**.**050**	0.61	**1**.**61 (1**.**00–2**.**58)**	**0**.**048**	0.38	
*ESR2*	rs1255998	G	1.16 (0.86–1.59)	0.33	**0**.**02**	1.28 (0.87–1.89)	0.20	0.20	0.95 (0.54–1.68)	0.86	**0**.**02**	
*ESR2*	rs928554	G	**0**.**70 (0**.**53–0**.**93)**	**0**.**013**	0.50	**0**.**64 (0**.**45–0**.**90)**	**0**.**013**	0.73	0.77 (0.47–1.27)	0.31	0.41	
*ESR2*	rs1271572	T	**0**.**67 (0**.**49–0**.**91)**	**0**.**011**	0.12	**0**.**52 (0**.**36–0**.**76)**^**§**^	**0**.**00074**	0.06	1.28 (0.70–2.33)^**§**^	0.42	0.33	
*FcγR3A*	rs396991	C	0.85 (0.64–1.12)	0.24	0.53	1.02 (0.72–1.44)	0.93	0.26	**0**.**47 (0**.**27–0**.**81)**	**0**.**0067**	0.80	
*SHBG*	rs6259	A	1.08 (0.79–1.48)	0.62	0.29	**1**.**55 (1**.**03–2**.**31)**	**0**.**033**	0.26	**0**.**48 (0**.**28–0**.**83)**	**0**.**0087**	0.14	

Abbreviations: SNP, single nucleotide polymorphism; OR, odds ratio; CI, confidence interval.

Data on RF was available in 422 RA patients in the replication population. Estimates were adjusted for age and sex. P ≤ 0.05 in bold.

The RF-specific effect modification of steroid hormone SNPs was determined by logistic regression using RF as interaction term.

Meta-analysis was conducted following a fixed effect model.

^†^Estimates calculated according to a dominant model of inheritance.

^**§**^Estimates calculated according to a recessive model of inheritance.

Similarly, we also found a RF-specific effect of the *CYP2C9*_rs1799853_ and *CYP1B1*_rs10012_ SNPs to determine the risk of developing bone erosions. Seropositive patients carrying the *CYP2C9*_rs1799853T/T_ or *CYP1B1*_rs10012G/G_ genotypes had a significantly reduced chance of developing bone erosions (OR = 0.16, *P* = 0.0007 and OR = 0.42, *P* = 0.0040) whereas an opposite but not significant effect was observed in seronegative patients (OR = 2.71, *P* = 0.23; *P*_Int_ = 0.003 and OR = 1.70, *P* = 0.35; *P*_Int_ = 0.031; Table 3). The effect of the *CYP2C9*_rs1799853_ polymorphism on the risk of developing erosive disease in seropositive patients remained statistically significant after correction for multiple testing (P < 0.00074), which suggested a role of the *CYP2C9* gene in modulating disease progression in RA. In accordance with these findings, we found that seropositive patients carrying the *CYP2C9*_AT_ haplotype had a significantly decreased risk of developing erosive disease (OR_RF+_ = 0.61, *P* = 0.0075) whereas no effect was observed in seronegative patients (OR_RF−_ = 0.87, *P* = 0.57; Supplementary Table 2). No significant anti-CCP effect modification was found for *CYP2C9* and *CYP1B1* variants to determine the appearance of bone erosions (*P*_Int_ = 0.88 and *P*_Int_ = 0.27) underlying the importance of considering RF when evaluating the impact of the *CYP2C9* and *CYP1B1* loci on the risk of developing erosive disease. Importantly, when we attempted to validate the RF-specific association of the *CYP1B1*_rs10012G/G_ genotype with a decreased risk of having erosive disease in the replication population, we found that seropositive patients carrying the *CYP1B1*_rs10012G/G_ genotype had a significantly decreased risk of developing bone erosions (OR_RF+_ = 0.30, *P* = 0.0051) whereas an opposite but not significant effect was found in seronegative patients (OR_RF−_ = 5.97, *P* = 0.10; *P*_Int_ = 0.012; Table 4). The meta-analysis of both populations confirmed the strong RF-specific effect of this SNP to determine the risk of developing bone erosions (OR_RF+_ = 0.38, *P* = 0.000081; *P*_Het_ = 0.52 vs. OR_RF−_ = 2.22, *P* = 0.11; *P*_Het_ = 0.31). Although we attempted to validate the association of the *CYP2C9*_rs1799853T/T_ genotype with a decreased risk of having erosive disease, the relatively small size of the replication population did not allow us to perform the association analysis according to a recessive model of inheritance. However, we found a RF-specific effect on the risk of having erosive disease for a neighboring SNP (rs1057910), which suggested a RF-dependent effect of the *CYP2C9* locus to modulate the risk of erosive disease (OR_RF+_ = 2.75, *P* = 0.027 vs. OR_RF−_ = 0.54, *P* = 0.47; Table 4). The meta-analysis of both cohorts confirmed the RF-specific effect of the *CYP2C9*_rs1057910_ SNP on the risk of developing bone erosions (OR_RF+_ = 2.68, *P* = 0.0022 vs. OR_RF−_ = 1.08, *P* = 0.83; *P*_Het_ = 0.34; Table 4).

In line with these findings, we also observed an additional RF effect modification of the *FcγR3A*_rs396991_ and *SHBG*_rs6259_ SNPs to determine the risk of having erosions. Seronegative patients carrying the *FcγR3A*_rs396991C_ allele had a significantly reduced chance of developing bone erosions (OR = 0.45, *P* = 0.013) whereas an opposite but not significant effect was observed in seropositive patients (OR = 1.18, *P* = 0.46; *P*_Int_ = 0.028; Table 3). Furthermore, seropositive subjects carrying the *SHBG*_rs6259A_ allele showed an increased risk of developing bone erosions (OR_RF+_ = 1.87, *P* = 0.015) whereas an opposite but not significant effect was detected in seronegative patients (OR_RF−_ = 0.66, *P* = 0.19). Interestingly, although the effect was stronger in seronegative patients, we could validate the RF-specific effect of the *SHBG*_rs6259_ SNP on the risk of developing erosions in the replication population (OR_RF+_ = 1.17, *P* = 0.63 vs. OR_RF−_ = 0.22, *P* = 0.009; *P*_Int_ = 0.013; Table 4) and the meta-analysis of both cohorts confirmed that the effect of this marker was dependent on the RF status (OR_RF+_ = 1.55, *P* = 0.033 vs. OR_RF−_ = 0.48, *P* = 0.0087; *P*_Het_ = 0.14; Table 4). Although we could not validate the RF-specific association of the *FcγR3A*_rs396991_ SNP with bone erosions in the DREAM registry, the meta-analysis of both cohorts confirmed the RF-specific effect of this SNP to modulate the risk of developing erosive disease (OR_RF−_ = 0.47, *P* = 0.0067 vs. OR_RF+_ = 1.02, *P* = 0.93). None of these two SNPs showed a significant effect modification by anti-CCP (*P*_Int_ = 0.85) suggesting again that RF, rather than anti-CCP, is a driver factor influencing the impact of the steroid hormone-related loci on disease progression in RA.

Finally, an overall association analysis revealed that carriers of the *ESR1*_rs1801132G_ allele showed a decreased risk of developing bone erosions (OR = 0.71, *P* = 0.034). Although we could not validate this association in the replication population, we found that this SNP showed a significant RF-specific effect to modulate the risk of developing bone erosions but according to a recessive model of inheritance. Thus, seropositive carriers of the *ESR1*_rs1801132G/G_ genotype showed a decreased risk of developing bone erosions (OR_RF+_ = 0.39, *P* = 0.004) whereas an opposite but not statistically significant effect was observed in seronegative subjects (OR_RF−_ = 1.43, *P* = 0.57; Table 4). Furthermore, we found a similar RF-specific effect for the *ESR1*_rs9340799_ SNP that was not detected in the discovery population (OR_RF+_ = 0.42, *P* = 0.009 vs OR_RF−_ = 8.33, *P* = 0.011). Considering that none of these associations survived after correction for multiple testing and that the effect of *ESR1* SNPs on the risk of developing erosive disease seemed to depend on the inheritance model applied, these results suggested a complex relationship between the *ESR1* locus and bone erosion probably mediated by more than one SNP. In support of this notion, we found that 3 large *ESR1* haplotypes (*ESR1*_CTATTTTCTA_, *ESR1*_CTATTCTTCA_, and ESR1_GTATTCTCTA_) were significantly associated with a decreased risk of having erosive disease (*P* = 0.0094, *P* = 0.0021 and *P* = 0.0023, respectively; Supplementary Table 2).

### Correlation of selected SNPs with steroid hormone levels

**Figure 1:**
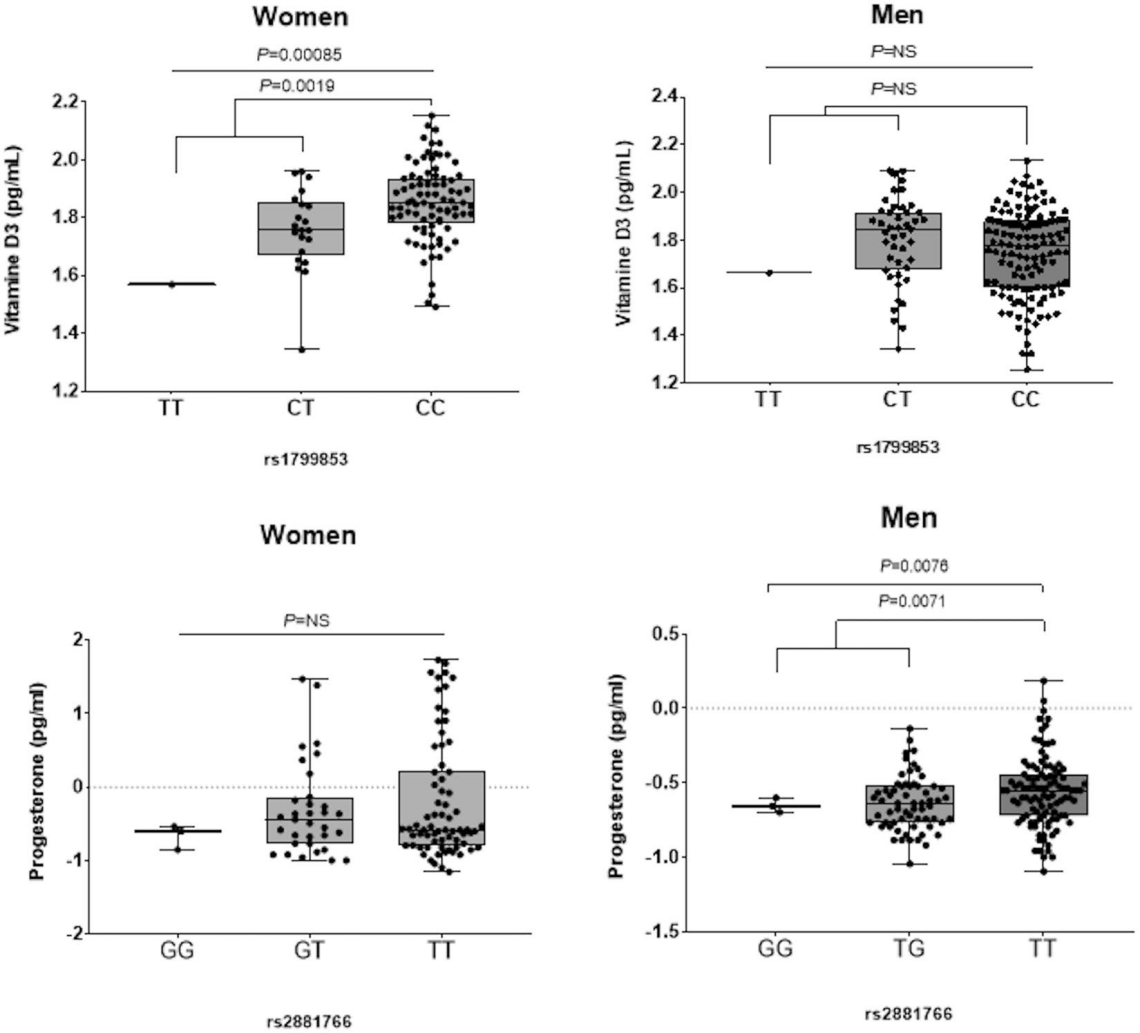
Correlation of the *CYP2C9*_rs1799853_ and *ESR1*_rs2881766_ SNPs with vitamin D3 and progesterone levels in women (n = 107) and men (n = 172). Patients using oral contraceptives were excluded from the analysis. After log transformation, linear regression analyses were adjusted for age. NS; non-significant.

Besides the strong genetic association with erosive disease identified for the *CYP2C9*_rs1799853_ SNP and its known role in controlling the metabolism of a wide range of drugs (with the T allele acting as poor metabolizer), we found that this coding variant strongly correlated with serum vitamin D3 levels in women (*P* = 0.00085 and *P* = 0.0019; Fig. 1) whereas no effect was detected in men. Although the association of the *CYP2C9*_rs1799853_ SNP with reduced levels of vitamin D3 in women remained borderline significant, this finding suggested that this marker might have a role in the modulation of bone homeostasis and vitamin D3-mediated immune responses.

On the other hand, we found that the *ESR1*_rs2881766G/G_ genotype or G allele weakly correlated with progesterone levels (*P* = 0.0076 and *P* = 0.0071) and that the *ESR1*_rs851984,_ *ESR1*_rs2077647_, *ESR1*_rs2071454_, *ESR1*_rs3798577_ and *ESR1*_rs910416_ variants mapped among histone marks in several cell types including osteoblasts and a wide variety of immune cells.

### Correlation of steroid hormone SNPs with cytokine levels

**Figure 2:**
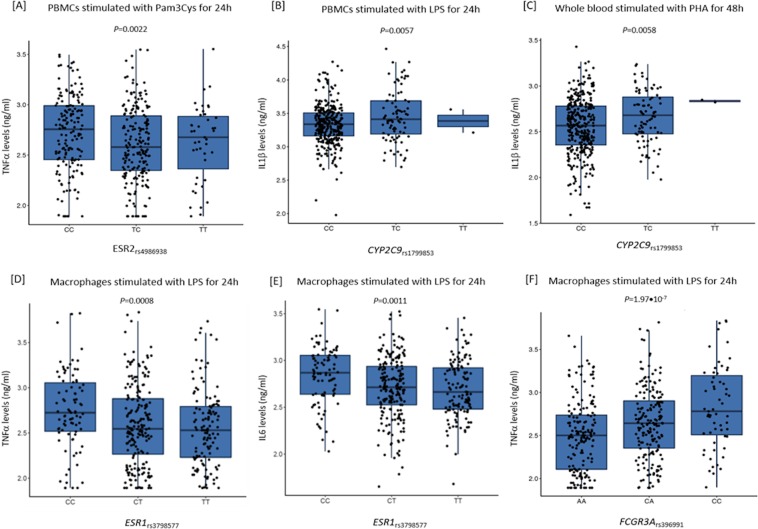
Correlation of hormone-related SNPs with cytokine levels after stimulation of PBMCs or macrophages with LPS, PHA or Pam3Cys (n = 408).

Interestingly, we also found that carriers of the *ESR2*_rs4986938T_ allele had reduced levels of TNFα after the stimulation of PBMCs with Pam3Cys for 24 h (*P* = 0.0022; Fig. 2A). These results along with those reporting that the *ESR2*_rs4986938_ and *ESR2*_rs1271572_ SNPs map among histone marks in multiple cell types including osteoblasts and different subsets of immune cells, suggest a possible functional role of the *ESR2* variants in modulating the risk of developing bone erosions likely through the modulation of ESR2 expression. In addition, we found that the presence of the *CYP2C9*_rs1799853T_ allele correlated with an increased production of IL1β after the stimulation of PBMCs with LPS or PHA for 24 h or 48 h (*P* = 0.0057 and *P* = 0.0058; Fig. 2B,C), which also pointed to a functional role of this marker in determining the presence of bone erosions.

In addition, we found that the *ESR1*_rs3798577_ variant correlated with TNFα and IL6 levels after the stimulation of human macrophages with LPS for 24 h (*P* = 0.00083 and 0.0011; Fig. 2D,E). Finally, we found that carriers of the *FcγR3A*_rs396991C_ allele showed a significantly increased production of TNFα after stimulation of macrophages with LPS for 24 h (*P* = 1.97•10^−7^; Fig. 2F). Of note, the association of the *FcγR3A*_rs396991C_ allele with increased levels of TNFα in macrophages survived after correction for multiple testing, which strongly suggested a functional effect of this variant to modulate macrophage-mediated immune responses, a key factor influencing the risk of developing erosive disease. On the contrary, although it was tempting to speculate that *ESR1*, *ESR2*, *CYP2C9* SNPs might also exert their effect on the risk of developing erosive disease through the modulation of steroid hormones or steroid hormone-mediated immune responses, it is important to mention that none of the associations between *ESR1*, *ESR2* or *CYP2C9* SNPs and cytokine levels survived after correction for multiple testing, which suggested a modest functional impact of these polymorphisms on the risk of developing bone erosions.

### Usefulness of steroid hormone-related SNPs to predict erosive disease

**Figure 3:**
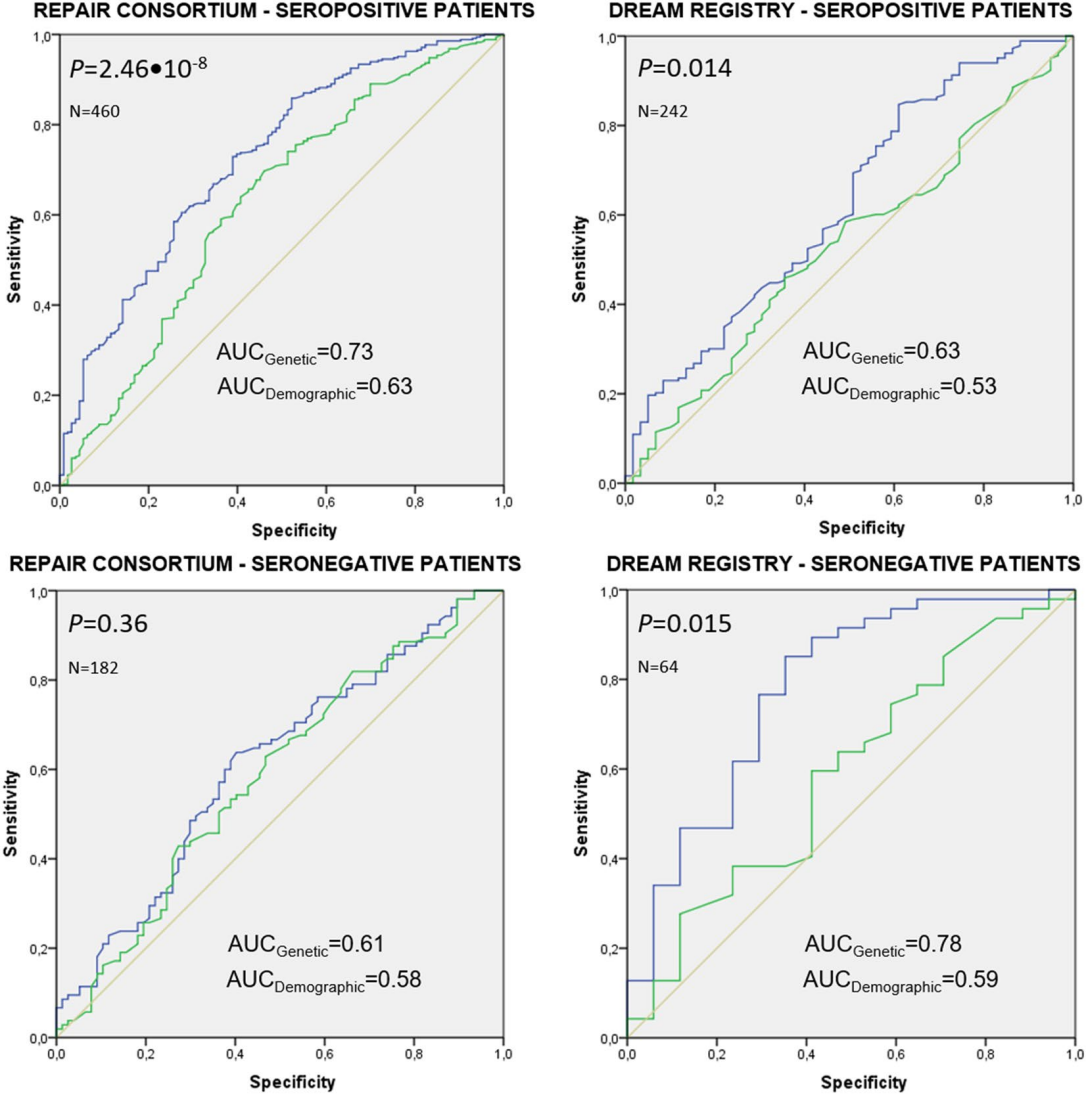
Receiver operating characteristics (ROC) curve analysis in the discovery and replication populations. ROC curves summarize the accuracy of prediction for genetic and demographic models in seropositive and seronegative patients. The genetic models (marked in blue) included SNPs that were significantly associated with erosive disease in seropositive patients (either in the single-SNP or haplotype analyses) whereas the demographic models included demographic variables (age and gender as covariates; marked in green) for seropositive and seronegative patients.

As a whole, our data suggest that the attributable effect of the *CYP1B1*, *CYP2C9*, *ESR1*, *ESR2*, *SHBG*, and *FcγR3A* loci to modulate the risk of developing bone erosions in RA patients might be dependent on the presence of either missense or intronic polymorphisms that affect the immune responses to a greater or lesser extent. Considering the strength of the RF-specific associations found for SNPs within *CYP1B1*, *CYP2C9*, *SHBG*, *ESR1*, *ESR2*, *FcγR3A* and *CYP3A4* loci in the discovery and/or replication populations, we decided to assess whether SNPs within these loci could be useful to differentially predict disease progression in seropositive and seronegative patients. Our results showed that the addition of 5 steroid hormone-related SNPs within the *CYP1B1*, *CYP2C9*, *CYP3A4*, *ESR2* and *SHBG* loci to a model including demographic variables significantly improved the ability to predict the appearance of bone erosions in seropositive patients (AUC_Genetic_ = 0.73 vs. AUC_Demographic_ = 0.63; *P* = 2.46•10^−8^; Table 5) whereas no significant predictive value was found for these SNPs in seronegative patients (AUC_Genetic_ = 0.61 vs. AUC_Demographic_ = 0.59; *P* = 0.36; Fig. 3). The consistency of this predictive analysis was confirmed through a permutation test that showed that none of the 50.000 permuted models for each group showed a better prediction capacity than the genetic model (Average sorted AUC = 0.644, Z-score = 6.79 and P_Z_score (50.000perm)_ = 5.67•10^−12^). Even though the lack of patients carrying the *CYP2C9*_rs1799853T/T_ genotype and the relatively small size of the replication population hampered the validation of this 5-SNP model to predict erosive disease, we attempted to confirm the utility of this model in the DREAM registry. We found that a similar model slightly improved the ability to predict erosive disease in both seropositive and seronegative patients (AUC_Genetic-RF+_ = 0.63 vs. AUC_Demographic-RF+_ = 0.53; *P* = 0.014 and AUC_Genetic-RF−_ = 0.78 vs. AUC_Demographic-RF−_ = 0.54; *P* = 0.015; Fig. 3). Despite these interesting results, only the *CYP1B1* and *CYP2C9* SNPs seemed to have a consistent predictive value for the development of bone erosions in seropositive patients.

**Table 5:** Discriminative value *AUC* for the model including estrogen-related variants in the discovery and replication populations.

Discovery population (REPAIR Consortium; n = 816; RF += 571 and RF− = 238)					Replication population (DREAM registry; n = 436; RF += 328 and RF− = 94)				
Demographic model (RF + Patients; n = 460)				LR test p-value	Demographic model (RF + Patients; n = 242)				LR test p-value
	P-value	OR 95%CI	AUC 95%CI^a^			P-value	OR 95%CI	AUC 95%CI^a^	
Gender (male)	**0**.**004**	**0**.**486 (0**.**296–0**.**798)**			Gender (male)	0.740	1.113 (0.590–2.100)		
Age	**0**.**00066**	**1**.**030 (1**.**013–1**.**048)**	0.629 (0.567–0.692)		Age	0.654	0.995 (0.972–1.018)	0.528 (0.445–0.612)	
**Predictive model including 5 SNPs (RF** + **patients; n** = 460)*					**Predictive model including 4 SNPs (RF** + **patients; n** = **242)Ϯ**				
	**P-value**	**OR 95%CI**	**AUC 95%CI** ^**a**^			**P-value**	**OR 95%CI**	**AUC 95%CI** ^**a**^	
*ESR2*_rs1271572_	**0**.**002**	**0**.**414 (0**.**236–0**.**726)**	0.730 (0.672–0.780)^‡^		*ESR2*_rs1271572_	0.763	0.899 (0.450–1.796)	0.625 (0.541–0.709)	
*CYP2C9*_rs1799853_	**0**.**024**	**0**.**226 (0**.**062–0**.**824)**			*CYP2C9*_rs1057910_	**0**.**058**	**3**.**385 (0**.**96–11**.**94)**		
*CYP1B1*_rs10012_	**0**.**013**	**0**.**442 (0**.**233–0**.**840)**			*CYP1B1*_rs10012_	**0**.**014**	**0**.**285 (0**.**105–0**.**772)**		
*CYP3A4*_rs2740574_	**0**.**005**	**5**.**793 (1**.**718–19**.**53)**			*CYP3A4*_rs2740574_	**0**.**075**	**0**.**370 (0**.**124–1**.**107)**		
*SHBG*_r6259_	**0**.**011**	**2**.**316 (1**.**212–4**.**425)**			*SHBG*_r6259_	0.444	1.378 (0.606–3.130)		
Gender (male)	**0**.**005**	**0**.**475 (0**.**281–0**.**803)**			Gender (male)	0.941	0.976 (0.505–1.884)		
Age	**0**.**00039**	**1**.**033 (1**.**015–1**.**052)**		**2**.**46•10**^**–8**^	Age	0.811	0.997 (0.972–1.022)		**0**.**014**
**Demographic model (RF− patients; n** = **182)**				**LR test p-value**	**Demographic model (RF− patients; n** = **64)**				**LR test p-value**
	**P-value**	**OR 95%CI**	**AUC 95%CI** ^**a**^			**P-value**	**OR 95%CI**	**AUC 95%CI** ^**a**^	
Gender (male)	0.713	0.864 (0.397–1.882)			Gender (male)	0.322	0.542 (0.161–1.820)		
Age	0.053	0.978 (0.956–1.000)	0.588 (0.503–0.672)		Age	0.511	1.014 (0.973–1.056)	0.590 (0.429–0.752)	
**Predictive model including 5 SNPs (RF− patients; n** = **182)**					**Predictive model including 4 SNPs (RF− patients; n** = **64)Ϯ**				
	**P-value**	**OR 95%CI**	**AUC 95%CI** ^**a**^			**P-value**	**OR 95%CI**	**AUC 95%CI** ^**a**^	
*ESR2*_rs1271572_	0.804	1.103 (0.509–2.388)	0.613 (0.530–0.696)		*ESR2*_rs1271572_	0.828	1.214 (0.212–6.937)	0.778 (0.640–0.917)	
*CYP2C9*_rs1799853_	0.105	6.052 (0.688–53.26)			*CYP2C9*_rs1057910_	0.219	0.287 (0.039–2.097)		
*CYP1B1*_rs10012_	0.521	1.526 (0.420–5.549)			*CYP1B1*_rs10012_	0.493	2.316 (0.209–25.60)		
*CYP3A4*_rs2740574_	0.422	0.668 (0.250–1.785)			*CYP3A4*_rs2740574_	**0**.**033**	**0**.**113 (0**.**015–0**.**836)**		
*SHBG*_r6259_	0.699	0.870 (0.43–1.760)			*SHBG*_r6259_	**0**.**029**	**0**.**139 (0**.**024–0**.**814)**		
Gender (male)	0.394	0.696 (0.303–1.600)			Gender (male)	0.287	0.453 (0.106–1.943)		
Age	0.065	0.979 (0.956–1.001)		0.36	Age	0.949	0.998 (0.950–1.049)		**0**.**015**

^a^Including age and gender as variables never dropped from models and when are compared with a baseline model with AUROC = 0.5. P ≤ 0.10 in bold (stepwise threshold).

*All SNPs showing a significant association with erosive disease (*P* < 0.10) were initially added to the model in the discovery population.

^‡^A sort analysis in the discovery population revealed that this model showed an AUC value systematically higher than those observed in 50.000 randomized models:

Average AUC of null distribution (50.000 models) = 0.644

Z score = 6.79, **P**_**Z_score-value_(50**.**000perm)**_** = 5**.**67**•**10**^**−12**^.

^Ϯ^All SNPs were forced to be included in the replication population with the exception of the *CYP2C9*_rs1057910_ that was included due to the impossibility to calculate association estimates for the *CYP2C9*_rs1799853_ SNP.

## Discussion

The present study reports, for the first time, both overall and RF-specific associations of steroid hormone-related polymorphisms with the risk of developing erosive RA. The most relevant effect was found for SNPs within *CYP1B1*, *CYP2C9*, and *ESR2* genes. We observed that seropositive RA patients carrying the *CYP1B1*_rs10012G/G_, *CYP2C9*_rs1799853T/T_, and *ESR2*_rs1271572T/T_, genotypes had a significantly reduced risk of developing bone erosions during the course of the disease whereas an opposite but not significant effect was found in seronegative patients. Although the relatively small size of the replication population hampered the validation of these associations according to a recessive model of inheritance, we could validate the RF-specific association of the *CYP1B1*_rs10012_ SNP with the risk of developing erosive disease in the replication population and the meta-analysis of the discovery and replication cohorts confirmed the strong RF effect modification of this SNP to determine the risk of bone erosions. In addition, although we could not validate the RF-specific association of the *CYP2C9*_rs1799853_ variant in the replication population due to the lack of patients carrying the *CYP2C9*_rs1799853T/T_ genotype, we found a RF-specific effect on the risk of having erosive disease for a neighbouring SNP within the *CYP2C9* locus (rs1057910) that was further confirmed through meta-analysis. Although this SNP was not in linkage disequilibrium (LD) with the rs1799853 and, therefore, does not represent the same association signal, these results support the idea that the *CYP2C9* locus might influence the risk of developing bone erosions in a RF dependent manner and likely through the modulation of the hormone metabolism and hormone-dependent immune responses.

Whilst the *CYP1B1* locus is located on chromosome 2p21-22, *CYP2C9* belongs to the *CYP2C* family, a gene cluster (*CYP2C19-CYP2C9-CYP2C8*) located on chromosome 10q23.3. Together with CYP1A2 and CYP3A4, CYP1B1 and CYP2C9 catalyze the conversion of estrogens to genotoxic catechol estrogens (estradiol 4- and 2-hydroxylation, respectively)^53^, which are key processes that allow the binding of catechol estrogens to ESR1 and ESR2. At low concentrations, CYP2C9 is also implicated in the 17beta-hydroxy dehydrogenation of estradiol creating estrone, which is one of the 3 natural estrogens with multiple immunomodulatory actions. Given that both the *CYP1B1*_rs10012_ and *CYP2C9*_rs1799853_ SNPs are coding variants that alters their respective protein amino acid sequences (R48G and Arg144Cys) and appear to decrease the activity of the enzyme but also proper folding and stability, it seems to be plausible to hypothesize that the presence of these SNPs could determinate estrogen-dependent immune responses and thereby modulate the risk of developing bone erosions. Our functional studies also demonstrated that the *CYP2C9*_rs1799853_ SNP correlated with serum vitamin D3 levels, which suggest that the *CYP2C9*_rs1799853_ SNP might also affect disease progression through the regulation of vitamin D3-mediated immune responses. However, we need to interpret these results with caution as we only found a significant correlation with vitamin D3 in women whereas no effect was seen in men. These results, together with those reporting that carriers of the *CYP2C9*_rs1799853T_ allele have an increased production of IL1β after the stimulation of PBMCs or whole blood with LPS or PHA, suggest that the protective effect attributed to this coding variant might not only depend on vitamin D3 but other factors such as RF or even other stimuli or substrates. In this regard, our team has reported that the *CYP2C9*_rs1799853_ polymorphism is strongly associated with poor response to anti-TNF drugs in RA^54^, suggesting that this missense variant might modulate the strength of immune responses through the regulation of the metabolism of endogenous compounds but also compounds administered exogenously.

Although we also attempted to validate the RF-specific association of the *ESR2*_rs1271572T/T_ genotype with a decreased risk of having erosion in the replication population, we only found a modest and not significant effect of this variant to determine erosive disease. However, the direction of the effect in seropositive and seronegative patients was similar to the one observed in the discovery population and the meta-analysis of both cohorts confirmed that the effect of this SNP on the risk of developing bone erosions was modified by RF. In support of a RF-specific effect of this variant to influence the risk of erosive RA, we found that seropositive carriers of the *ESR2*_CGTA_ haplotype had a decreased risk to develop erosive RA whereas no effect was detected in seronegative patients. Interestingly, an overall haplotype analysis also revealed a significant association of 3 common haplotypes within the *ESR1* locus (*ESR1*_CTATTTTCTA_, *ESR1*_CTATTCTTCA_, and ESR1_GTATTCTCTA_) with a decreased risk of developing bone erosions, which also pointed to a role of *ESR1* SNPs in modulating the risk of erosions.

The *ESR2* and *ESR1* genes (14q23.2 and 6q25.1 respectively) encode the estrogen receptor beta (ESRβ) and alpha (ESRα) that are highly expressed in synovial cells^55^ and bone^56^ but also in most of the immune cells^57^. Although a number of experimental studies have shown that female RA patients have worse prognosis and higher disease activity and health assessment questionnaire scores in comparison with male patients, it is also well established that steroid hormones have both pro- and anti-inflammatory roles in RA. Although it has been reported, for instance, that the activation of ESRs by estradiol (E_2_) often leads to joint protection and the maintenance of bone density (by inhibiting bone resorption)^58^ and that the withdrawal of estrogens drastically increases the severity of the disease (by promoting joint destruction, bone erosions and physical disability)^59^, it has been also reported that RA patients have high levels of estrone in the synovial fluid compared to healthy individuals and that estrogens can also induce pro-inflammatory responses through the activation of different mechanisms involving humoral immunity^60^, multiple transcription factors (such as c/EBPβ, STAT-1, NFκB) and oxidative stress pathways (especially those involving iNOs)^61,62^. Furthermore, it has been reported that estrogens are able to promote pro-inflammatory pathways including B- and T-cell proliferation^63^, thymocyte maturation^64^, cell trafficking^65^ and the expression of specific adhesion molecules^63^. Although the existing paradox with respect to the immunomodulating role of steroid hormones in RA remain unresolved, it seems to be reasonable to hypothesize that the presence of *ESR2* polymorphisms that correlate either with *ESR2* mRNA expression levels may influence on the risk of developing bone erosions in RA likely through the modulation of ESR2-dependent tolerogenic immune responses. In addition, although the association of the *ESR2* and *ESR1* polymorphisms with serum hormone levels or TNF and IL6 levels in stimulated macrophages did not remain significant after correction for multiple testing, our functional findings were in agreement with the genetic results suggesting a protective effect of *ESR2* polymorphisms and specific *ESR1* haplotypes on the risk of developing erosive RA. In addition, our genetic and functional results were also concordant with data of previous studies reporting that the presence of certain SNPs, microsatellites or even specific haplotypes within estrogen receptor genes is associated with bone mineral density and influences the risk of developing bone erosions^66,67^ affecting RA patients^68^ but also subjects diagnosed with other chronic inflammatory diseases^69^ and bone degenerative diseases^70,71^.

Finally, this study also showed a weak but still interesting RF-specific effect of the *SHBG*_rs6259_ and *Fc*γ*R3A*_rs396991_ SNPs to determine the risk of having erosions. Importantly, we could validate the RF-specific effect of the *SHBG*_rs6259_ SNP on the risk of developing erosions in the replication population and the meta-analysis of both cohorts confirmed that the effect of this marker was strongly dependent on the RF status. On the other hand, although the RF-specific association of the *FcγR3A*_rs396991_ SNP with bone erosions was not statistically significant in the DREAM registry, the meta-analysis of both cohorts confirmed that the effect of this SNP on the risk of bone erosions was modified by the RF.

Whereas little is known about the role of the *SHBG* locus (17p13) in determining RA progression, a number of experimental studies have shown that the *Fc*γ*R3A* locus (1q23) is involved in the recognition of IgG1 and IgG3 by NK cells and macrophages and that the activation of this receptor by IgG and IgG-RF immunocomplexes might lead to the initiation of a range of sustained and harmful inflammation events that, if chronified, may cause joint and bone destruction and promote the onset of RA^72–74^. In this context and considering the number of studies reporting association of the *FcγR3A*_rs396991_ SNP with autoimmune diseases^75–82^, the response to a wide range of biologic drugs^83–89^ and an exacerbated production of TNFα after stimulation of macrophages with LPS for 24 h but also the reported association of the *SHBG*_rs6259_ with serum SHBG levels^90^, we hypothesize that these SNPs might also play a relevant role in determining bone erosions and disease progression.

Considering the noticeable RF-specific impact of the *CYP1B1*, *CYP2C9*, *ESR2*, *FcγR3A* and *SHBG* SNPs on the risk of developing erosive disease but also their functional implication in modulating hormone levels and/or immune responses, we decided to assess if the presence of steroid hormone-related SNPs could be useful to reliably predict the appearance of bone erosions in seropositive and seronegative patients separately. To do that, we built a genetic model including demographic variables and those SNPs that were consistently associated with the risk of developing bone erosions in seropositive patients. After removing the SNPs that were not significantly associated with erosive disease in the model, we obtained a model including 5 SNPs within the *CYP1B1*, *CYP2C9*, *CYP3A4*, *ESR2*, and *SHBG* loci that significantly improved the ability to predict the risk of developing erosive disease when compared with a reference model including demographic variables. The predictive capacity of these SNPs was restricted to seropositive patients since the addition of the same SNPs (or any other genetic marker) to a model built with demographic variables in seronegative patients did not show any predictive value. The predictive ability of the genetic model in seropositive patients was consistent as no similar models were found after performing a 50.000 permutation test. When we attempted to confirm the utility of this model in the DREAM registry, we found that the *CYP1B1* and *CYP2C9* SNPs in seropositive patients showed a consistent predictive value for the development of bone erosions. These results suggest that *CYP1B1* and *CYP2C9* SNPs alone or in combination with other clinical and genetic markers might help to improve the ability to predict the appearance of bone erosions in seropositive patients (~70% of RA patients). Additional studies including these and other genetic and clinical markers are urgently needed to improve our ability to predict disease progression in RA.

This study has strengths and weaknesses. The strengths of this study include a relatively large and well-characterized population and the meta-analyses conducted considering results from the DREAM registry. In the discovery population, we had 80% of power to detect an odds ratio of 1.68 (α = 0.00074) for a SNP with a frequency of 0.25, which underlined the feasibility of the study design. Another important strength of this study is the development of cytokine stimulation experiments and the measurement of seven serum steroid hormones in a large cohort of healthy subjects, which allowed us to investigate the functional role of the most interesting markers in modulating immune responses but also to test their impact on determining steroid hormone levels. A drawback is the multicenter nature of this study that placed inevitable limitations such as the impossibility of using available scores to better define bone erosions (Sharp van der Heijde, Genant, SENSE, and Ratingen scores). Given the cross-sectional approach of the study, we had also intrinsic limitations such as a possible bias due to variations in treatments and follow-up time among study participants. Finally, it is important to mention that the selection of SNPs for this study was influenced by the limited research funds and that the relatively small size of the replication population hampered the validation of the most interesting associations that were detected when a recessive model of inheritance was assumed.

## Conclusion

In conclusion, this candidate gene association study suggests, for the first time, that the presence of *CYP1B1*, *CYP2C9*, *ESR1*, *ESR2*, *SHBG* and *FcγR3A* SNPs or haplotypes influence the risk of developing bone erosions in RA. This study also shows that the effect found for most of the SNPs or haplotypes was dependent on the RF status and that genotyping of hormone-related SNPs might help to reliably predict disease progression in seropositive patients.

## Supplementary information

Supplementary Figure 1

Supplementary Table 1

Supplementary Table 2

Supplementary Table 3
