# Supplementary material for: Steroid hormone-related polymorphisms associate with the development of bone erosions in rheumatoid arthritis and help to predict disease progression: Results from the REPAIR consortium
Source: Sci Rep. 2019 Oct 15;9:14812. doi: 10.1038/s41598-019-51255-0 (PMC6794376; doi:10.1038/s41598-019-51255-0)
Supplement: Supplementary file 1 — Supplementary Figure 1 [file 41598_2019_51255_MOESM1_ESM.docx]

**Steroid hormone-related polymorphisms associate with the development of bone erosions in rheumatoid arthritis and help to predict disease progression: Results from the REPAIR consortium**

Jose M. Sánchez-Maldonado^1,2^, Rafael Cáliz MD, PhD^1,2,3^, Luz Canet PhD^1^, Rob ter Horst^4^, Olivier Bakker PhD^5^, Alfons A den Broeder MD PhD^6^, Manuel Martínez-Bueno PhD^7^, Helena Canhão MD, PhD^8^, Ana Rodríguez Ramos^1^, Carmen B. Lupiañez PhD^1^, María José Soto-Pino^3^, Antonio García MD PhD^3^, Eva Pérez-Pampin MD PhD^9^, Alfonso González-Utrilla MD PhD^3^, Alejandro Escudero MD PhD^10^, Juana Segura-Catena^1^, Romana T. Netea-Maier PhD^4^, Miguel A. Ferrer MD PhD^3^, Eduardo Collantes-Estevez MD PhD^10^, Miguel Ángel López Nevot MD PhD^11^, Yang Li PhD^5^, Manuel Jurado^1,2^, João E. Fonseca MD PhD^12,13^, Mihai G. Netea MD PhD^4, 14^, Marieke J. H. Coenen PhD^15^, Juan Sainz PhD^1,2^

**Supplementary Figure 1.** Linkage disequilibrium (LD*)* blocks in the hormone-related genes calculated in the discovery population.


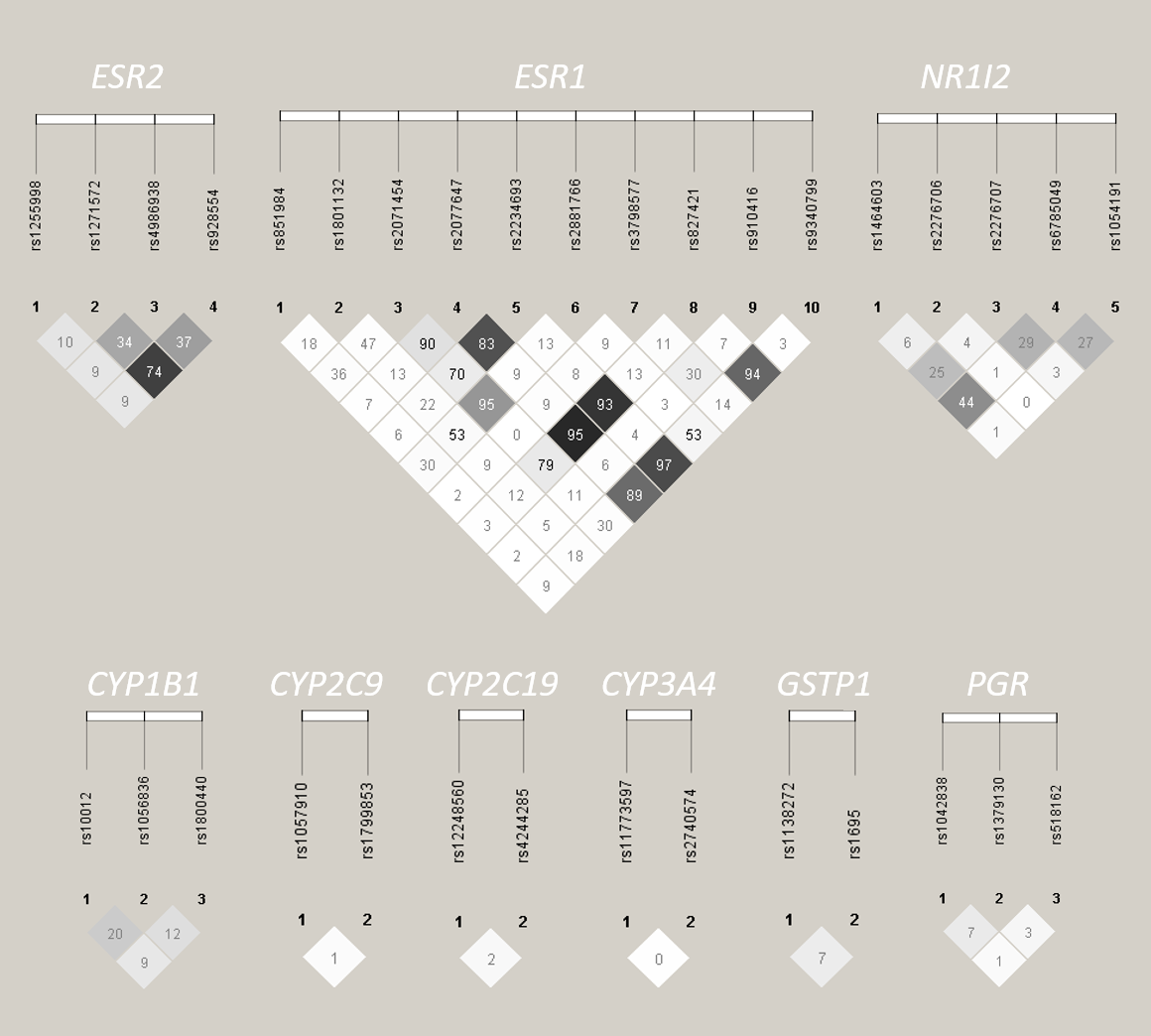


Numbers into squares indicate r^2^ values (dark color also represents the degree of LD).
