# Supplementary material for: Steroid hormone-related polymorphisms associate with the development of bone erosions in rheumatoid arthritis and help to predict disease progression: Results from the REPAIR consortium
Source: Sci Rep. 2019 Oct 15;9:14812. doi: 10.1038/s41598-019-51255-0 (PMC6794376; doi:10.1038/s41598-019-51255-0)
Supplement: Supplementary file 2 — Supplementary Table 1 [file 41598_2019_51255_MOESM2_ESM.docx]

**Steroid hormone-related polymorphisms associate with the development of bone erosions in rheumatoid arthritis and help to predict disease progression: Results from the REPAIR consortium**

Jose M. Sánchez-Maldonado^1,2^, Rafael Cáliz MD, PhD^1,2,3^, Luz Canet PhD^1^, Rob ter Horst^4^, Olivier Bakker PhD^5^, Alfons A den Broeder MD PhD^6^, Manuel Martínez-Bueno PhD^7^, Helena Canhão MD, PhD^8^, Ana Rodríguez Ramos^1^, Carmen B. Lupiañez PhD^1^, María José Soto-Pino^3^, Antonio García MD PhD^3^, Eva Pérez-Pampin MD PhD^9^, Alfonso González-Utrilla MD PhD^3^, Alejandro Escudero MD PhD^10^, Juana Segura-Catena^1^, Romana T. Netea-Maier PhD^4^, Miguel A. Ferrer MD PhD^3^, Eduardo Collantes-Estevez MD PhD^10^, Miguel Ángel López Nevot MD PhD^11^, Yang Li PhD^5^, Manuel Jurado^1,2^, João E. Fonseca MD PhD^12,13^, Mihai G. Netea MD PhD^4, 14^, Marieke J. H. Coenen PhD^15^, Juan Sainz PhD^1,2^

**Supplementary Table 1.** Overall and anti-CCP-specific associations of estrogen-related polymorphisms and risk of developing erosive disease.

| **Gene** | **SNP ID** | **Effect**  **allele** | **Overall (n=816)** | | **Anti-CCP+ patients (n=490)** | | **Anti-CCP- patients (n=183)** | |  |
| --- | --- | --- | --- | --- | --- | --- | --- | --- | --- |
|  |  |  | **OR (95% CI)**^†^ | ***P*** | **OR (95% CI)**^†^ | ***P*** | **OR (95% CI)**^†^ | ***P*** | ***P_Interaction_*** |
| *CYP1A1* | rs1799814 | A | 0.85 (0.52-1.39) | 0.52 | 0.78 (0.41-1.46) | 0.43 | 0.82 (0.30-2.24) | 0.69 | 0.78 |
| *CYP1A2* | rs762551 | C | 0.91 (0.66-1.25) | 0.57 | **0.58 (0.38-0.90)** | **0.016** | 1.34 (0.71-2.53) | 0.37 | **0.031** |
| *CYP1B1* | rs1800440 | G | 1.06 (0.76-1.46) | 0.74 | 0.96 (0.62-1.49) | 0.87 | 1.03 (0.51-2.07) | 0.94 | 0.95 |
| *CYP1B1* | rs1056836 | G | 0.92 (0.64-1.32) | 0.66 | 1.15 (0.71-1.86) | 0.57 | 0.55 (0.26-1.18) | 0.13 | 0.13 |
| *CYP1B1* | rs10012 | G | 1.05 (0.76-1.46) | 0.77 | 1.03 (0.66-1.61) | 0.89 | 1.55 (0.80-2.98) | 0.19 | 0.27 |
| *CYP2C9* | rs1799853 | T | **0.70 (0.51-0.98)** | **0.036** | **0.57 (0.36-0.88)** | **0.012** | 0.59 (0.30-1.15) | 0.12 | 0.88 |
| *CYP2C9* | rs1057910 | C | 1.68 (0.98-2.89) | 0.059 | 1.54 (0.73-3.22) | 0.26 | 1.46 (0.57-3.76) | 0.43 | 0.94 |
| *CYP2C19* | rs12248560 | T | 1.04 (0.75-1.44) | 0.83 | 1.29 (0.83-2.01) | 0.27 | **0.48 (0.23-0.99)** | **0.046** | **0.015** |
| *CYP2C19* | rs4244285 | A | 1.00 (0.69-1.44) | 0.99 | 0.95 (0.58-1.56) | 0.84 | 1.33 (0.60-2.95) | 0.48 | 0.38 |
| *CYP3A4* | rs2740574 | G | 1.57 (0.90-2.74) | 0.11 | **2.75 (1.12-6.74)** | **0.027** | 0.96 (0.33-2.78) | 0.94 | 0.13 |
| *CYP3A4* | rs11773597 | C | 1.19 (0.77-1.84) | 0.43 | 1.20 (0.65-2.20) | 0.56 | 1.61 (0.68-3.79) | 0.28 | 0.48 |
| *CYP17A1* | rs743572 | G | 0.92 (0.66-1.28) | 0.63 | 0.95 (0.61-1.47) | 0.82 | 0.77 (0.37-1.57) | 0.47 | 0.69 |
| *ESR1* | rs851984 | T | 1.07 (0.78-1.46) | 0.68 | 1.01 (0.66-1.55) | 0.96 | 1.23 (0.65-2.31) | 0.53 | 0.66 |
| *ESR1* | rs2881766 | G | 1.00 (0.72-1.39) | 0.99 | 1.04 (0.66-1.63) | 0.86 | 0.73 (0.37-1.42) | 0.35 | 0.38 |
| *ESR1* | rs2071454 | G | 0.96 (0.65-1.42) | 0.82 | 1.11 (0.64-1.92) | 0.71 | 0.57 (0.26-1.24) | 0.16 | 0.18 |
| *ESR1* | rs2077647 | G | 0.92 (0.65-1.30) | 0.64 | 0.63 (0.39-1.01) | 0.056 | 1.73 (0.89-3.37) | 0.11 | **0.011** |
| *ESR1* | rs827421 | C | 0.95 (0.67-1.33) | 0.75 | 0.72 (0.45-1.16) | 0.18 | 1.55 (0.79-3.05) | 0.20 | **0.050** |
| *ESR1* | rs2234693 | C | 1.10 (0.78-1.55) | 0.58 | 0.84 (0.52-1.35) | 0.50 | 1.82 (0.91-3.63) | 0.088 | 0.051 |
| *ESR1* | rs9340799 | G | 0.97 (0.71-1.34) | 0.87 | 0.82 (0.53-1.27) | 0.38 | 1.75 (0.91-3.34) | 0.091 | **0.043** |
| *ESR1* | rs1801132 | G | **0.71 (0.52-0.97)** | **0.034** | 0.70 (0.46-1.08) | 0.11 | 0.68 (0.36-1.32) | 0.26 | 0.84 |
| *ESR1* | rs3798577 | C | 1.21 (0.87-1.68) | 0.27 | 1.51 (0.97-2.35) | 0.068 | 1.07 (0.54-2.14) | 0.84 | 0.46 |
| *ESR1* | rs910416 | T | 0.84 (0.59-1.19) | 0.33 | 0.91 (0.57-1.44) | 0.67 | 0.73 (0.36-1.51) | 0.40 | 0.56 |
| *ESR2* | rs1255998 | G | 0.92 (0.64-1.33) | 0.67 | 1.15 (0.69-1.91) | 0.60 | 0.78 (0.36-1.70) | 0.54 | 0.45 |
| *ESR2* | rs928554 | G | 0.82 (0.66-1.02) | 0.077 | 0.65 (0.41-1.02) | 0.063 | 0.98 (0.50-1.89) | 0.94 | 0.31 |
| *ESR2* | rs4986938 | A | 1.09 (0.79-1.51) | 0.59 | 1.13 (0.72-1.75) | 0.60 | 1.02 (0.52-2.00) | 0.96 | 0.82 |
| *ESR2* | rs1271572 | T | **0.55 (0.37-0.82)^§^** | **0.004** | **0.47 (0.27-0.82)^§^** | **0.008** | 1.05 (0.46-2.37)**^§^** | 0.91 | 0.11 |
| *FCGR2A* | rs1801274 | G | 1.04 (0.72-1.51) | 0.82 | 0.82 (0.49-1.36) | 0.43 | 1.44 (0.65-3.20) | 0.37 | 0.23 |
| *FCGR3A* | rs396991 | C | 0.90 (0.64-1.27) | 0.56 | 0.90 (0.56-1.43) | 0.64 | 0.94 (0.47-1.90) | 0.87 | 0.85 |
| *GSTP1* | rs1695 | G | 1.05 (0.77-1.44) | 0.76 | 0.99 (0.65-1.52) | 0.97 | 0.68 (0.36-1.29) | 0.24 | 0.35 |
| *GSTP1* | rs1138272 | T | 1.32 (0.77-2.25) | 0.31 | 1.74 (0.82-3.69) | 0.15 | 0.38 (0.10-1.45) | 0.16 | 0.073 |
| *HSD17B1* | rs605059 | T | 1.12 (0.79-1.59) | 0.54 | 1.26 (0.78-2.04) | 0.35 | 1.06 (0.67-1.68) | 0.80 | 0.63 |
| *NR1I2* | rs2276706 | A | 1.01 (0.74-1.40) | 0.93 | 0.91 (0.59-1.42) | 0.68 | 0.96 (0.49-1.87) | 0.91 | 0.80 |
| *NR1I2* | rs1464603 | C | 1.16 (0.85-1.59) | 0.35 | 1.12 (0.73-1.72) | 0.59 | 1.15 (0.61-2.16) | 0.67 | 0.97 |
| *NR1I2* | rs6785049 | G | 0.92 (0.66-1.27) | 0.60 | 0.88 (0.57-1.36) | 0.56 | 1.13 (0.58-2.21) | 0.73 | 0.62 |
| *NR1I2* | rs2276707 | T | 1.03 (0.73-1.45) | 0.87 | 0.83 (0.53-1.31) | 0.43 | 1.76 (0.83-3.70) | 0.14 | 0.11 |
| *NR1I2* | rs1054191 | A | 0.41 (0.17-1.00)**^§^** | 0.049 | **0.26 (0.09-0.80)^§^** | **0.019** | 0.47 (0.06-3.80)**^§^** | 0.48 | 0.87 |
| *PGR* | rs1042838 | A | 0.85 (0.62-1.17) | 0.32 | 0.90 (0.56-1.48) | 0.70 | **0.42 (0.21-0.86)** | **0.018** | 0.071 |
| *PGR* | rs1379130 | A | 0.92 (0.66-1.26) | 0.59 | 1.34 (0.87-2.07) | 0.19 | 0.91 (0.47-1.77) | 0.79 | 0.32 |
| *PGR* | rs518162 | A | 1.11 (0.75-1.64) | 0.62 | 1.12 (0.67-1.89) | 0.66 | 1.08 (0.48-2.40) | 0.86 | 0.96 |
| *SHBG* | rs6259 | A | 1.22 (0.83-1.78) | 0.31 | **1.80 (1.04-3.13)** | **0.036** | 0.81 (0.40-1.66) | 0.57 | 0.054 |
| *SULT1A1* | rs9282861 | A | 1.04 (0.76-1.43) | 0.79 | 1.00 (0.65-1.53) | 0.99 | 1.14 (0.60-2.17) | 0.70 | 0.61 |

Abbreviations: SNP, single nucleotide polymorphism; OR, odds ratio; CI, confidence interval; Anti-CCP, antibodies to cyclic citrullinated peptide. Data on anti-CCP was available in 673 RA patients. Estimates were adjusted for age, sex and country of origin. P<0.05 in bold.

^†^ Estimates calculated according to a dominant model of inheritance.

**^§^** Estimates calculated according to a recessive model of inheritance.
