# Supplementary material for: Steroid hormone-related polymorphisms associate with the development of bone erosions in rheumatoid arthritis and help to predict disease progression: Results from the REPAIR consortium
Source: Sci Rep. 2019 Oct 15;9:14812. doi: 10.1038/s41598-019-51255-0 (PMC6794376; doi:10.1038/s41598-019-51255-0)
Supplement: Supplementary file 3 — Supplementary Table 2 [file 41598_2019_51255_MOESM3_ESM.docx]

**Steroid hormone-related polymorphisms associate with the development of bone erosions in rheumatoid arthritis and help to predict disease progression: Results from the REPAIR consortium**

Jose M. Sánchez-Maldonado^1,2^, Rafael Cáliz MD, PhD^1,2,3^, Luz Canet PhD^1^, Rob ter Horst^4^, Olivier Bakker PhD^5^, Alfons A den Broeder MD PhD^6^, Manuel Martínez-Bueno PhD^7^, Helena Canhão MD, PhD^8^, Ana Rodríguez Ramos^1^, Carmen B. Lupiañez PhD^1^, María José Soto-Pino^3^, Antonio García MD PhD^3^, Eva Pérez-Pampin MD PhD^9^, Alfonso González-Utrilla MD PhD^3^, Alejandro Escudero MD PhD^10^, Juana Segura-Catena^1^, Romana T. Netea-Maier PhD^4^, Miguel A. Ferrer MD PhD^3^, Eduardo Collantes-Estevez MD PhD^10^, Miguel Ángel López Nevot MD PhD^11^, Yang Li PhD^5^, Manuel Jurado^1,2^, João E. Fonseca MD PhD^12,13^, Mihai G. Netea MD PhD^4, 14^, Marieke J. H. Coenen PhD^15^, Juan Sainz PhD^1,2^

**Supplementary Table 2.** Haplotype association analysis for erosive disease in RA patients stratified by RF.

| **ESR1** | **rs1801132** | **rs2071454** | **rs2077647** | **rs2234693** | **rs2881766** | **rs3798577** | **rs827421** | **rs851984** | **rs910416** | **rs9340799** | **Freq** | **RA patients**  **(n=816)**  **OR (95% CI)** | ***P*** | **Freq** | **RF+patients**  **(n=571)**  **OR (95% CI)** | ***P*** | **Freq** | **RF- patients**  **(n=238)**  **OR (95% CI)** | ***P*** |
| --- | --- | --- | --- | --- | --- | --- | --- | --- | --- | --- | --- | --- | --- | --- | --- | --- | --- | --- | --- |
| **1** | C | T | A | T | T | C | T | C | C | A | 0.0872 | 1.00 | --- | 0.0822 | 1.00 | --- | 0.0996 | 1.00 | --- |
| **2** | C | T | G | C | T | T | C | C | T | G | 0.0680 | 0.42 (0.17 - 1.06) | 0.065 | 0.0750 | 0.54 (0.20 - 1.50) | 0.24 | 0.0358 | 0.77 (0.12 - 5.01) | 0.78 |
| **3** | C | T | A | T | T | T | T | C | C | A | 0.0462 | 0.36 (0.11 - 1.20) | 0.096 | 0.0537 | 1.69 (0.32 - 8.86) | 0.53 | 0.0115 | 0.09 (0.00 - 1.73) | 0.11 |
| **4** | C | T | G | C | T | C | C | C | C | G | 0.0434 | 0.82 (0.24 - 2.78) | 0.74 | 0.0457 | 0.89 (0.23 - 3.36) | 0.86 | 0.0331 | 0.86 (0.08 - 9.47) | 0.9 |
| **5** | C | T | A | T | T | T | T | C | T | A | 0.0432 | **0.25 (0.09 - 0.71)** | **0.0094** | 0.0449 | **0.10 (0.03 - 0.40)** | **0.001** | 0.0421 | 0.40 (0.07 - 2.36) | 0.32 |
| **6** | G | T | A | T | T | T | T | C | T | A | 0.0405 | 0.58 (0.21 - 1.62) | 0.3 | 0.0367 | 0.75 (0.19 - 3.00) | 0.69 | 0.0324 | 0.48 (0.10 - 2.27) | 0.35 |
| **7** | C | T | A | T | T | T | T | T | T | A | 0.0362 | 0.41 (0.13 - 1.26) | 0.12 | 0.0420 | 0.32 (0.08 - 1.33) | 0.12 | 0.0501 | 3.78 (0.51 - 28.1) | 0.2 |
| **8** | C | T | G | C | T | T | C | T | T | G | 0.0349 | 1.35 (0.32 - 5.68) | 0.68 | 0.0421 | 0.59 (0.17 - 2.07) | 0.41 | 0.0123 | 0.06 (0.00 - 2.91) | 0.16 |
| **9** | C | T | A | T | T | T | T | T | C | A | 0.0324 | 3.92 (0.48 - 32.0) | 0.2 | 0.0304 | 0.82 (0.12 - 5.42) | 0.83 | 0.0313 | 0.23 (0.03 - 1.74) | 0.16 |
| **10** | C | T | A | T | T | C | T | T | C | A | 0.0301 | **0.12 (0.03 - 0.47)** | **0.0021** | 0.0271 | 1.04 (0.16 - 6.62) | 0.96 | 0.0398 | **0.02 (0.00 - 0.24)** | **0.0033** |
| **11** | C | T | G | C | T | T | C | T | C | G | 0.0283 | **0.21 (0.07 - 0.70)** | **0.011** | 0.0299 | **0.23 (0.06 - 0.88)** | **0.033** | 0.0244 | 4.16 (0.27 - 63.2) | 0.31 |
| **12** | C | T | A | T | T | C | T | T | T | A | 0.0262 | 4.43 (0.48 - 40.8) | 0.19 | 0.0187 | na (na-na) | na | 0.0218 | na (na-na) | na |
| **13** | C | T | A | T | G | T | T | C | T | A | 0.0248 | 0.69 (0.22 - 2.13) | 0.52 | 0.0278 | 0.49 (0.13 - 1.83) | 0.29 | --- | na (na-na) | na |
| **14** | C | T | G | C | T | C | C | T | C | G | 0.0238 | 1.03 (0.26 - 4.03) | 0.97 | 0.0229 | 0.24 (0.05 - 1.08) | 0.064 | --- | na (na-na) | na |
| **15** | C | G | G | C | G | C | C | C | T | A | 0.0212 | 0.95 (0.27 - 3.31) | 0.93 | 0.0252 | 1.06 (0.25 - 4.44) | 0.94 | --- | na (na-na) | na |
| **16** | C | T | G | C | T | T | C | C | C | G | 0.0210 | 1.10 (0.16 - 7.51) | 0.92 | 0.0161 | 1.49 (0.06 - 34.3) | 0.80 | 0.0541 | 0.74 (0.16 - 3.40) | 0.7 |
| **17** | C | T | A | T | T | C | T | C | T | A | 0.0190 | 0.34 (0.05 - 2.19) | 0.26 | 0.0281 | **0.18 (0.04 - 0.82)** | **0.026** | 0.0179 | 0.35 (0.01 - 11.36) | 0.56 |
| **19** | G | T | G | C | T | C | C | C | C | G | 0.0162 | 0.26 (0.07 - 1.01) | 0.052 | --- | na (na-na) | na | --- | na (na-na) | na |
| **18** | C | T | G | C | T | C | C | T | T | G | 0.0152 | **0.15 (0.03 - 0.81)** | **0.027** | --- | na (na-na) | na | 0.0348 | 0.86 (0.11 - 6.89) | 0.89 |
| **20** | G | T | A | T | T | C | T | T | C | A | 0.0147 | **0.14 (0.03 - 0.75)** | **0.022** | 0.0196 | **0.12 (0.02 - 0.60)** | **0.010** | 0.0157 | 0.38 (0.03 - 4.93) | 0.46 |
| **21** | G | T | A | T | T | C | T | C | T | A | 0.0130 | **0.07 (0.01 - 0.38)** | **0.0023** | --- | na (na-na) | na | 0.0259 | 0.24 (0.04 - 1.36) | 0.11 |
|  |  |  |  |  |  |  |  |  |  |  |  |  |  |  |  |  |  |  |  |
| **CYP2C9** | rs1057910 | rs1799853 | 99999 |  |  |  |  |  |  |  | **Freq** | RA patients  (n=816)  OR (95% CI) | *P* | Freq | RF+patients  (n=571)  OR (95% CI) | *P* | Freq | RF- patients  (n=238)  OR (95% CI) | *P* |
| **1** | A | C |  |  |  |  |  |  |  |  | 0.7742 | 1.00 | --- | 0.7755 | 1.00 | --- | 0.7644 | 1.00 | --- |
| **2** | A | T |  |  |  |  |  |  |  |  | 0.1660 | **0.72 (0.54 - 0.96)** | **0.024** | 0.1706 | **0.61 (0.43 - 0.88)** | **0.0075** | 0.1597 | 0.87 (0.53 - 1.42) | 0.57 |
| **3** | C | C |  |  |  |  |  |  |  |  | 0.0593 | 1.48 (0.89 - 2.45) | 0.13 | 0.053 | 2.09 (0.96 - 4.56) | 0.063 | 0.0759 | 1.21 (0.60 - 2.44) | 0.6 |
|  |  |  |  |  |  |  |  |  |  |  |  |  |  |  |  |  |  |  |  |
| **ESR2** | rs1255998 | rs1271572 | rs4986938 | rs928554 |  |  |  |  |  |  | **Freq** | RA patients  (n=816)  OR (95% CI) | *P* | Freq | RF+ patients  (n=571)  OR (95% CI) | *P* | Freq | RF- patients  (n=238)  OR (95% CI) | *P* |
| **1** | C | T | C | G |  |  |  |  |  |  | 0.3665 | 1.00 | --- | 0.3702 | 1.00 | --- | 0.3562 | 1.00 | --- |
| **2** | C | G | T | A |  |  |  |  |  |  | 0.3638 | **1.30 (1.01 - 1.68)** | **0.042** | 0.3636 | **1.63 (1.20 - 2.25)** | **0.0051** | 0.3667 | 0.93 (0.62-1.40) | 0.99 |
| **3** | G | G | C | A |  |  |  |  |  |  | 0.1342 | 1.12 (0.80 - 1.57) | 0.52 | 0.1294 | 1.39 (0.90 - 2.16) | 0.14 | 0.1436 | 0.80 (0.46-1.40) | 0.53 |
| **4** | C | G | C | A |  |  |  |  |  |  | 0.0658 | 1.65 (0.95 - 2.85) | 0.074 | 0.0728 | **2.05 (1.02 - 4.12)** | **0.043** | 0.0510 | 0.94 (0.36-2.50) | 0.98 |
| **5** | C | G | C | G |  |  |  |  |  |  | 0.0187 | 1.26 (0.52 - 3.03) | 0.61 | 0.0169 | 0.95 (0.30 - 3.04) | 0.93 | 0.0223 | 1.29 (0.34-4.90) | 0.63 |
| **6** | C | T | T | A |  |  |  |  |  |  | 0.0186 | 1.09 (0.42 - 2.86) | 0.86 | 0.0198 | 1.11 (0.34 - 3.69) | 0.86 | 0.0141 | 0.72 (0.11-4.80) | 0.84 |
| **7** | C | T | C | A |  |  |  |  |  |  | 0.0185 | 0.80 (0.34 - 1.93) | 0.63 | 0.0114 | 0.53 (0.12 - 2.33) | 0.4 | 0.0338 | 1.10 (0.37-3.20) | 0.78 |

**^†^** Estimates calculated according to a dominant model and adjusted for age, sex and country of origin.

Minimum haplotype frequency was set at 0.01. P<0.05 in bold.
