# Supplementary material for: Steroid hormone-related polymorphisms associate with the development of bone erosions in rheumatoid arthritis and help to predict disease progression: Results from the REPAIR consortium
Source: Sci Rep. 2019 Oct 15;9:14812. doi: 10.1038/s41598-019-51255-0 (PMC6794376; doi:10.1038/s41598-019-51255-0)
Supplement: Supplementary file 4 — Supplementary Table 3 [file 41598_2019_51255_MOESM4_ESM.docx]

**Steroid hormone-related polymorphisms associate with the development of bone erosions in rheumatoid arthritis and help to predict disease progression: Results from the REPAIR consortium**

Jose M. Sánchez-Maldonado^1,2^, Rafael Cáliz MD, PhD^1,2,3^, Luz Canet PhD^1^, Rob ter Horst^4^, Olivier Bakker PhD^5^, Alfons A den Broeder MD PhD^6^, Manuel Martínez-Bueno PhD^7^, Helena Canhão MD, PhD^8^, Ana Rodríguez Ramos^1^, Carmen B. Lupiañez PhD^1^, María José Soto-Pino^3^, Antonio García MD PhD^3^, Eva Pérez-Pampin MD PhD^9^, Alfonso González-Utrilla MD PhD^3^, Alejandro Escudero MD PhD^10^, Juana Segura-Catena^1^, Romana T. Netea-Maier PhD^4^, Miguel A. Ferrer MD PhD^3^, Eduardo Collantes-Estevez MD PhD^10^, Miguel Ángel López Nevot MD PhD^11^, Yang Li PhD^5^, Manuel Jurado^1,2^, João E. Fonseca MD PhD^12,13^, Mihai G. Netea MD PhD^4, 14^, Marieke J. H. Coenen PhD^15^, Juan Sainz PhD^1,2^

**Supplementary Table 3.** ESR2 polymorphisms are ESR2 eQTLs

| ESR2 dbSNPs | Position | Reference Allele | Alternative  Allele | Tissue | Normalized Effect Size | ESR2 expression *P*-value |
| --- | --- | --- | --- | --- | --- | --- |
| rs1271572 | Near gene | A | G | Skin (sun exposed and unexposed) | Negative | 8.32e-23 / 2.6e-25 / 3.8e-9 |
|  |  | A | G | Lymphoblastoid_EUR_exonlevel | Negative | 3.47e-10 |
|  |  | A | G | Muscle Skeletal | Negative | 6.60e-6 |
|  |  | A | G | Nerve Tibial | Negative | 2.60e-5 |
|  |  | A | G | Whole blood | Negative | 1.98e-06 / 3.1e-9 |
| rs928554 | 3’-UTR | C | T | Skin (sun exposed and unexposed) | Negative | 4.08e-31 / 2.4e-41 / 9.5e-15 |
|  |  | C | T | Lymphoblastoid_EUR_exonlevel | Negative | 8.90e-15 |
|  |  | C | T | Muscle Skeletal | Negative | 6.90e-8 |
|  |  | C | T | Nerve Tibial | Negative | 3.70e-6 |
|  |  | C | T | Thyroid | Negative | 4.5e-7 |
|  |  | C | T | Lung | Negative | 8.20e-7 |
|  |  | C | T | Whole blood | Negative | 9.75e-08 |
| rs4986938 | 3’-UTR | C | T | Skin (sun exposed) | Negative | 3.85e-08 / 5.6e-13 |
|  |  | C | T | Nerve Tibial | Negative | 5.60e-8 |
| rs1255998 |  | C | T | Lymphoblastoid_EUR_exonlevel | Negative | 5.03e-06 |
|  |  |  |  |  |  |  |

Data extracted from GTEx portal (https://www.gtexportal.org/home/) and/or haploreg (<http://archive.broadinstitute.org/mammals/haploreg/haploreg.php>).

A negative effect size indicate that the tested allele is associated with a decreased mRNA expression of the gene.
